# MLR-predictor: a versatile and efficient computational framework for multi-label requirements classification

**DOI:** 10.3389/frai.2024.1481581

**Published:** 2024-11-27

**Authors:** Summra Saleem, Muhammad Nabeel Asim, Ludger Van Elst, Markus Junker, Andreas Dengel

**Affiliations:** ^1^Department of Computer Science, Rheinland Pfälzische Technische Universität, Kaiserslautern, Germany; ^2^German Research Center for Artificial Intelligence (DFKI), Kaiserslautern, Germany

**Keywords:** software requirements, multi-label requirements, OkapiBM25, swarm optimizer, label powerset, data transformation, machine learning classifiers, deep learning predictors

## Abstract

**Introduction:**

Requirements classification is an essential task for development of a successful software by incorporating all relevant aspects of users' needs. Additionally, it aids in the identification of project failure risks and facilitates to achieve project milestones in more comprehensive way. Several machine learning predictors are developed for binary or multi-class requirements classification. However, a few predictors are designed for multi-label classification and they are not practically useful due to less predictive performance.

**Method:**

MLR-Predictor makes use of innovative OkapiBM25 model to transforms requirements text into statistical vectors by computing words informative patterns. Moreover, predictor transforms multi-label requirements classification data into multi-class classification problem and utilize logistic regression classifier for categorization of requirements. The performance of the proposed predictor is evaluated and compared with 123 machine learning and 9 deep learning-based predictive pipelines across three public benchmark requirements classification datasets using eight different evaluation measures.

**Results:**

The large-scale experimental results demonstrate that proposed MLR-Predictor outperforms 123 adopted machine learning and 9 deep learning predictive pipelines, as well as the state-of-the-art requirements classification predictor. Specifically, in comparison to state-of-the-art predictor, it achieves a 13% improvement in macro F1-measure on the PROMISE dataset, a 1% improvement on the EHR-binary dataset, and a 2.5% improvement on the EHR-multiclass dataset.

**Discussion:**

As a case study, the generalizability of proposed predictor is evaluated on softwares customer reviews classification data. In this context, the proposed predictor outperformed the state-of-the-art BERT language model by F-1 score of 1.4%. These findings underscore the robustness and effectiveness of the proposed MLR-Predictor in various contexts, establishing its utility as a promising solution for requirements classification task.

## 1 Introduction

**Figure 1:**
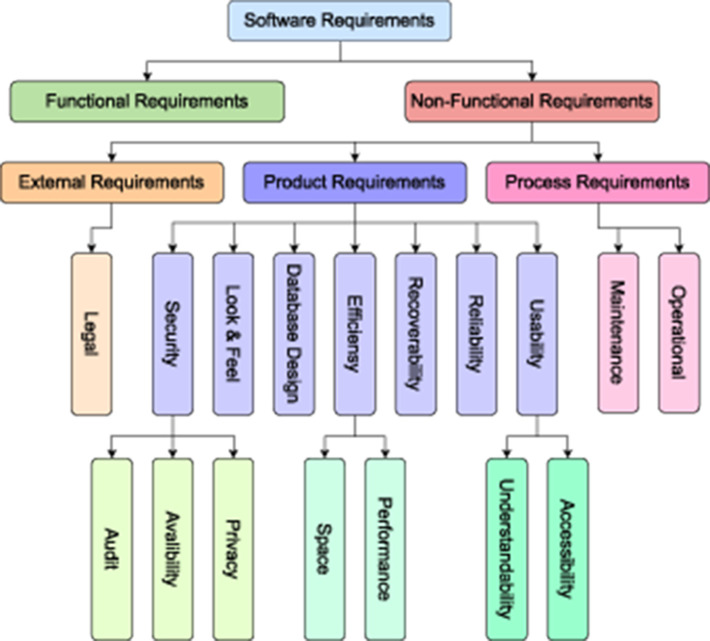
Hierarchical illustration of requirements classification.

To create a prosperous software application, usually software development teams including researchers and developers follow particular software development model such as waterfall (Petersen et al., 2009), v-Model (Ruparelia, 2010), agile (Zhang and Patel, 2010), spiral (Boehm, 1988), and incremental model (Larman and Basili, 2003). In all these models, requirements classification and understanding is common and fundamental task (Munassar and Govardhan, 2010). It is impossible to develop a successful software without completely understanding end users' requirements (Vogelsang and Borg, 2019; Hidellaarachchi et al., 2021). Requirements provide essential insights about features such as functionality and characteristics of the software that stakeholders expect and need (Gupta et al., 2020). To understand required features of a software, requirements classification is an indispensable task. Figure 1 illustrates requirements class hierarchy in which each class represents a unique aspect or feature of software, such as, functional class requirements represents information about capabilities and functionalities that software is expected to perform (Maruping and Matook, 2020). Similarly, non-functional requirements class defines qualities and constraints of a software. Moreover, sub-classes of this category represent a unique feature such as speed, reliability, performance, throughput, privacy, scalability, and security (Becker et al., 2019; Horkoff, 2019; Binkhonain and Zhao, 2019).

Manual categorization of requirements is time-consuming, expensive, and tedious task (Leelaprute and Amasaki, 2022). With an aim to automate the process of requirements classification, several rule-based predictors have been designed (Vlas and Robinson, 2011, 2012; Singh et al., 2016). However, requirements vary from software to software, and rule-based predictors are less generic as they require amendments in rules according to nature of software requirements (Vlas and Robinson, 2012). To overcome these problems, there has been a marathon of utilizing artificial intelligence methods power for developing more accurate and robust predictors capable of categorizing requirements into predefined classes (Hey et al., 2020; Althanoon and Younis, 2021; Dias Canedo and Cordeiro Mendes, 2020; Kaur and Kaur, 2022).

According to working paradigm, existing requirements classification predictors can be categorized into three different classes: binary (Tiun et al., 2020; Kurtanović and Maalej, 2017), multiclass (Khayashi et al., 2022; Ajagbe and Zhao, 2022; Kici et al., 2021), and multi-label (AlDhafer et al., 2022; Chen et al., 2022). Binary classification type-related predictors categorize requirements into functional and non-functional classes (Abad et al., 2017; Hey et al., 2020). Multiclass classification type-based predictors categorize requirements into one of the many predefined classes (Tóth and Vidács, 2019). Similarly, multi-label classification type-specific predictors categorize requirements into multiple classes, where same requirement may belong to multiple classes at the same time. Among all three classification types, multi-label classification of requirements is more useful as it comprehend different characteristics of software simultaneously. Primarily, it captures various dimensions of a requirement that cannot be adequately expressed through single class. This broader perspective allows for more nuanced analysis and representation of software's features and attributes.

According to the best of our knowledge, in last 4 years, five different predictors have been proposed for binary classification (Althanoon and Younis, 2021; Dias Canedo and Cordeiro Mendes, 2020; Rahimi et al., 2020; Tiun et al., 2020; Saleem et al., 2023), six for multiclass classification (Tóth and Vidács, 2019; Haque et al., 2019; Kaur and Kaur, 2022; Kici et al., 2021; Baker et al., 2019; Rahman et al., 2019) and seven for both binary and multiclass classification (Khayashi et al., 2022; Hey et al., 2020; Li et al., 2022; Rahimi et al., 2021; Ajagbe and Zhao, 2022; Fávero and Casanova, 2021; Luo et al., 2022). However to data, for multi-label classification, only three predictors have been proposed (AlDhafer et al., 2022; Slankas and Williams, 2013; Rashwan et al., 2013). Among all three classification types, multi-label classification predictors have least predictive performance. This is primarily because requirements classification across multiple labels is more challenging task in comparison with binary or multiclass classification (Gargiulo et al., 2018). In binary classification, functional class requirements typically encompass distinct features that differentiate them from non-functional class requirements. Machine learning predictors leverage these distinguishing patterns to classify requirements into functional and non-functional categories. In contrast, for multiclass classification, individual classes of requirements often share fewer distinctive features, leading to challenges in predictor performance. Similarly, in multi-label classification, the similarity among diverse classes of requirements is more pronounced, resulting in limited discriminative potential. Another major factor contributing to the lower predictive performance lies in the relatively small size of requirements datasets, especially in the context of multi-label classification. This issue is particularly pronounced as the distribution of training samples becomes smaller, making it challenging to accurately predict and classify multiple labels for the given requirements.

Considering industrial need for a powerful multi-label classification predictor for automatic requirements classification, the article in hand presents a versatile computational framework named MLR-Predictor. The presented framework contains diverse types of methods that together make multiple end-to-end predictive pipelines for multi-label requirements classification. A primary contribution of this article is to enrich framework with a unique method Okapi BM25 that transforms requirements text into statistical vectors by assigning weights to words based on their discriminative potential. This method has been widely used in the domain of information retrieval for transforming queries and document text into statistical vectors. For the very first time, we have introduced this method in the domain of text classification. Apart from this encoder, we also incorporated word2vec and FastText based pre-trained word embeddings. Moreover, we strengthened framework with four algorithm adaptation-based methods (multi-label k-nearest neighbor, binary relevance k-nearest neighbor, twin multi-label support vector machine, Multi-Label Hierarchical ARAM Neural Network), three data transformation methods [binary relevance (BR), label powerset (LP), and classifier chain (CC)], and nine traditional classifiers [support vector machine (SVM; Tong and Koller, 2001), naive Bayes (NB; Watkins, 1989), logistic regression (LR; LaValley, 2008), adaptive boosting (AdaBoost; Margineantu and Dietterich, 1997), random forest (RF; Breiman, 2001), decision tree (DT; Quinlan, 1996), gradient boosting (GB; Friedman, 2021), extreme gradient boosting (XGB; Chen et al., 2015), and extra tree (ET; Geurts et al., 2006)]. With an aim to design best predictive pipelines by utilizing each method with optimal set of hyper-parameters, framework is enriched with swarm optimizer that facilitates smart strategy for finding hyper-parameters best combination values. To compare proposed framework predictive pipeline performance, apart from state-of-the-art requirement classification predictors, we adapt nine different deep learning predictors that have been widely utilized in diverse types of text classification tasks. We perform a large-scale experimentation over three public benchmark datasets to find suitable answers of following research questions:

1. Which combination of traditional data transformation technique and machine learning (ML) classifier is most effective in designing predictive pipeline capable of accurately annotating requirements with their relevant classes?
2. Can OkapiBM25 generates more comprehensive and informative statistical vectors than TF-IDF and word2vec as well as FastText based word embeddings?
3. Do classifiers and OkapiBM25 hyper-parameters optimization through Particle Swarm Optimizer (PSO) enhance predictive pipelines performance?
4. Utilizing traditional data transformation and algorithm adaptation methods, is it possible to develop a generic predictive pipeline for multi-label requirements classification?
5. Do generic pre-trained word embeddings have potential for enhancing deep learning predictors performance for requirements classification?
6. While dealing with small datasets of requirements, which type of predictors yields superior performance for multi-label classification: deep learning predictors or machine learning predictors?

## 2 Related work

To cope with the challenges of software requirement analysis, researchers are trying to explore the potential of machine and deep learning approaches. The primary objective is to leverage the capabilities of these methods to assist software developers and analysts in creating comprehensive software requirement specifications (SRS) by systematically categorizing requirements into predefined classes. This section summarizes diverse types of machine and deep learning predictors that have been proposed for binary or multiclass and multi-label classification of requirements.

Among existing functional and non-functional requirements classification predictors (Althanoon and Younis, 2021; Dias Canedo and Cordeiro Mendes, 2020; Rahimi et al., 2020; Tiun et al., 2020; Saleem et al., 2023), one predictor (Althanoon and Younis, 2021) made use of TFIDF representation with two standalone machine learning classifiers, namely, Multinomial Naive Bayes (MNB) and logistic regression (LR). One predictor (Rahimi et al., 2020) used TFIDF representation and reaped combine potential of five machine learning classifiers, namely, logistic regression (LR), support vector classifier (SVC), support vector machine (SVM), decision tree (DT), and naive bayes (NB). Two predictors (Dias Canedo and Cordeiro Mendes, 2020; Saleem et al., 2023) utilized filter-based feature selection approaches along with four machine learning classifiers: LR, SVM, MNB, k-nearest neighbor (KNN), and attention-based deep learning classifier. One predictor (Tiun et al., 2020) explored potential of FastText and word2vec-based pre-trained word embeddings and convolutional neural network (CNN) architecture. Furthermore, among existing studies for multiclass classification of requirements (Haque et al., 2019; Tóth and Vidács, 2019; Kaur and Kaur, 2022; Kici et al., 2021; Baker et al., 2019; Rahman et al., 2019), two predictors (Haque et al., 2019; Tóth and Vidács, 2019) used TFIDF representation with 12 different standalone machine learning classifiers [MNB, SVM, Stochastic Gradient Descent SVM (SGD SVM), Bernoulli Naive Bayes (BNB), Gaussian Naive Bayes (GNB), KNearest Neighbors (KNN), DT, ET, Label propagation, Label spread, LR, MLP]. Two predictors (Kaur and Kaur, 2022; Rahman et al., 2019) investigated potential of glove, word2vec pre-trained embeddings with recuurent neural network (RNN) variants. One predictor (Baker et al., 2019) used random embeddings with artificial neuarl network (ANN) and CNN. One predictor (Kici et al., 2021) explored potential of five language models, namely, BERT, AL-BERT, Roberta, DistilBERT, and XLNet.

Within the realm of both binary and multiclass requirements classification, two predictors (Rahimi et al., 2021; Khayashi et al., 2022) studied effectiveness of deep learning-based meta-predictors. Second, five predictors (Fávero and Casanova, 2021; Hey et al., 2020; Li et al., 2022; Ajagbe and Zhao, 2022; Luo et al., 2022) explored potential of BERT language model. On the other hand, for multi-label requirements classification (AlDhafer et al., 2022; Slankas and Williams, 2013; Rashwan et al., 2013), one predictor (Slankas and Williams, 2013) made use of TFIDF representation method along with binary relevance-based data transformation approach and SVM classifier, while other predictor (AlDhafer et al., 2022) investigated integer encoding representation with bidirectional gated recurrent unit (BiGRU). One predictor (Rashwan et al., 2013) used ontologies with SVM classifier. Apart from requirements classification, for other software related tasks such as multi-label classification of users reviews about softwares, two predictors (Kaur and Kaur, 2023; Jha and Mahmoud, 2019) explored binary relevance-based data transformation along with SVM and BERT model. Table 1 summarizes aforementioned predictors for requirements classification in terms of dataset, feature encoding and classifier.

**Table 1:** Comprehensive summary of existing research studies for requirements classification.

**Classification type**	**References**	**Dataset**	**Feature encoding technique**	**Predictor**
Binary	Saleem et al., 2023	Promise, Promise-exp	FastText	Attention-based DL Classifier
	Althanoon and Younis, 2021	Promise	-	MNB, LR
	Tiun et al., 2020	Promise	Word2vec, Fasttext	CNN
	Dias Canedo and Cordeiro Mendes, 2020	Promise-exp	BoW, TF-IDF	LR, SVM, KNN, MNB
	Rahimi et al., 2020	Self-collected	TF-IDF	Ensemble of SVM, SVC, LR, NB, DT
Multiclass	Kaur and Kaur, 2022	Promise, Open source project	Glove	Self-attention-based bidirectional RNN
	Rahman et al., 2019	Promise	Word2Vec	RNN, LSTM, GRU
	Baker et al., 2019	Promise	Random word embeddings	ANN, CNN
	Haque et al., 2019	Promise	BoW, TF-IDF	NB, SVM, DT, KNN
	Tóth and Vidács, 2019	Promise, Stack overflow dataset	TF-IDF	BernoulliNB, DT ET, ETs, KNN, Label propagation, Label spread, LR, MLP, MNB, SVM
	Kici et al., 2021	Doors, Promise, Pure	Pre-trained BERT Model	BERT, DistillBERT, Roberta, Al-BERT, XLNet
Binary and multiclass	Khayashi et al., 2022	Promise, NFR-review, NFR-so	Pre-trained BERT Model	Prompt learning using BERT
	Luo et al., 2022	Pure	Glove, Keras word embeddings	CNN, LSTM, BiLSTM, GRU, BiGRU
	Li et al., 2022	Promise, Concordia	Node embedding	Graph attention network
	Ajagbe and Zhao, 2022	Promise, Pure, App review dataset, Google play store reviews	Pre-trained BERT Model	BERT
	Rahimi et al., 2021	Promise	Random word embeddings	Ensemble learning using CNN, LSTM, BiLSTM, GRU
	Fávero and Casanova, 2021	Open source project	BERT based embeddings	BERT
	Hey et al., 2020	Promise	Pre-trained BERT Model	BERT
Multi-label	AlDhafer et al., 2022	PromiseML, EHR-Binary EHR-Multiclass	Integer encoding	BiGRU
	Rashwan et al., 2013	Concordia	Unigram tokens with Ontology	SVM
	Slankas and Williams, 2013	PromiseML, EHR-Multiclass	BoW, TF-IDF	SVM

## 3 Materials and methods

This section briefly describes different methods that are incorporated in the proposed framework. It illustrates details of deep learning predictors that are adapted for requirements classification. Furthermore, it summarizes diverse types of evaluation measures that are used to evaluate and compare performance of proposed framework predictive pipelines with adapted deep learning predictors and state-of-the-art predictors. Finally, it describes details of public benchmark datasets.

### 3.1 Proposed MLR framework

**Figure 2:**
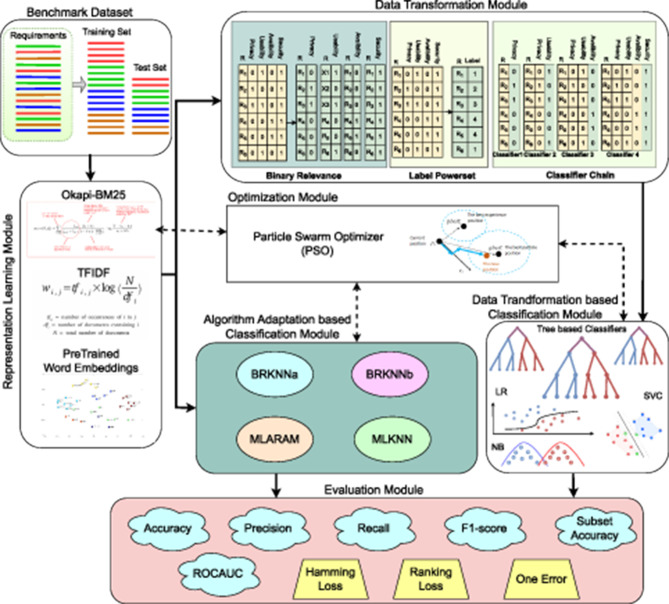
Graphical illustration of proposed MLR framework.

Figure 2 provides a visual representation of proposed framework different modules that facilitates development of versatile multi-label classification predictive pipelines. It can be seen in Figure 2 first of all representation learning module transforms requirements text into statistical vectors. This module is a fundamental component of the predictive pipelines because classifiers inherently rely on statistical vectors as they are unable to operate directly on raw text data. After transforming requirements text into statistical vectors, data transformation module transforms multi-label data into multiclass or binary classification data that is further passed to traditional machine learning classifiers.

On the other hand, in case of algorithm adaption-based predictive pipelines, representation learning module output is directly passed to algorithm adaptation module that performs classification. In other words, framework facilitates development of requirements classification predictive pipelines in two different ways either using representation learning module, data transformation, and classification module or by using representation learning module along with algorithm adaption-based classification module. Moreover, optimization module is optional as it can be utilized to find optimal hyper-parameters of different methods present in three different modules, namely, representation learning, classification, and algorithm adaption-based classification. Following subsections briefly describe details of these modules.

#### 3.1.1 Representation learning module

This module facilitates two traditional representation learning methods, namely, TF-IDF and OkapiBM25 encoder that is adapted from information retrieval domain. Furthermore, following success of pre-trained word embeddings in diverse types of natural language processing tasks including question answering system (Abbasiantaeb and Momtazi, 2021), biological sequence classification (Ao et al., 2022; Abu-Qasmieh et al., 2023), fake news detection (Verma et al., 2021; Agarwal et al., 2020), and medical code category assignment (Lin et al., 2019), we enriched representation learning module with two pre-trained word embeddings (word2vec, FastText) provided by kutuzov et al..^1^ Primarily authors (Kutuzov et al., 2017) generated both types of word embeddings by training word2vec and FastText models over large English wikipedia and English CoNLL17 corpus (Kutuzov et al., 2017). Pre-trained word embeddings enabled deep learning predictors to perform better even on small datasets. However, in requirements multi-label classification, potential of these embeddings remained unexplored. This research unlock the potential of pre-trained word embeddings for requirements multi-label classification task.

##### 3.1.1.1 TF-IDF

TF-IDF method transforms requirements text into statistical feature space by computing words term frequencies and inverse document frequencies (Ramos, 2003). The term frequency (TF) refers to occurrence count of a word *w* in requirement *R*. Document frequency (DF) showcases the count of corpus requirements in which word *w* appears. Inverse document frequency (IDF) of word *w* can be determined through Equation 1.


(1) $$\begin{array}{l} IDF=log\frac{n}{DF_{w}} \end{array}$$

In Equation 1, *n* refers to corpus total number of requirements and *DF*_*w*_ denotes document frequency of word *w*. Equation 2 illustrates mathematical expression for computing TF-IDF is score of word *w* with respect to requirement *R*.


(2) $$\begin{array}{l} TF-IDF_{w,R}=TF_{w,R}\cdot IDF_{w} \end{array}$$

The TF-IDF method assigns scores to words within the range of 0 to 1, where a word score approaching 1 indicates frequent occurrence and *vice versa*.

##### 3.1.1.2 OkapiBM25

OkapiBM25 encoder has been widely used in information retrieval domain for transforming quires and documents text into statistical vectors (Kanapala et al., 2019; Desai et al., 2022; Yu, 2019; Bokhari et al., 2021). This research explores the potential of OkapiBM25 encoder for transforming requirements text into statistical vectors. This encoder transforms requirements text into statistical vectors by computing words term and inverse document frequencies along with two special hyper-parameters (k, b). Equation 3 illustrates mathematical expression for computing term frequency of word *w* in requirement *R*.


(3) $$\begin{array}{l} TF\left(w,R\right)=\frac{TF}{\left[TF+k\times \left(1-b+b\times \frac{RL}{ARL}\right)\right]} \end{array}$$

In Equation 3, *TF* refers to term frequency of word w in requirement R. *RL* represents length of requirement *R*, and *ARL* denotes average length of requirements. *k* hyper-parameter controls terms frequencies saturation and hyper-parameter *b* minimizes the impact of requirements length variability. Length of requirements influences occurrence frequencies of words; hence, it is important to regularize occurrence frequencies with respect to requirements lengths. To understand this concept, consider two requirements: *R*_1_ and *R*_2_ comprising of 100 and 200 words, respectively. A word “w” appears 5 times in *R*_1_ and 10 times in *R*_2_. Although, *R*_2_ length is twice that of R1 but without considering their length variability word “w” gets double score in *R*_2_ compared to its score in *R*_1_. This factor hinders incorporation of real discriminative patters in generated feature space. In this study, hyper-parameter “B” optimal value assigns appropriate score to word “w” in both requirements.

Equation 4 depicts mathematical illustration of OkapiBM25 encoder for computing inverse document frequency of a word *w*.


(4) $$IDF(w)=\frac{log((N-n(w)+0.5)}{\left(n(w)+0.5)+1\right)}$$

In Equation 4, *n* refers to corpus total number of requirements, and *n*(*w*) denotes number of requirements containing word *w*.

Equation 5 illustrates mathematical expression for computing OkapiBM25 score of word w.


(5) $$\begin{array}{l} \text{OkapiBM25}(w,R)=\;\left(\frac{TF}{\left[TF+k\times (1-b+b\times \frac{RL}{ARL})\right]}\right)\\ \;\times \;\left(\frac{log((N-n(w)+0.5)}{\left(n(w)+0.5)+1\right)}\right) \end{array}$$

#### 3.1.2 Data transformation module

Data transformation methods transform multi-label data into single-label data and leverage binary or multiclass classifiers to perform classification. Over the time, researchers have proposed several data transformation approaches including binary relevance (BR; Boutell et al., 2004), label powerset (LP; Tsoumakas and Vlahavas, 2007), ranking by pairwise comparison (RPC; Hüllermeier et al., 2008), calibrated ranking by pairwise comparison (CRPC; Fürnkranz et al., 2008), and classifier chains (CC; Read et al., 2011). The proposed framework is enriched with three most widely used data transformation methods (BR, LP, CC) that are briefly described in following subsections.

##### 3.1.2.1 Binary relevance

BR (Boutell et al., 2004) transforms multi-label classification dataset into L binary classification datasets, where L represents number of unique labels in multi-label classification dataset. To briefly understand data transformation process, consider a multi-label classification dataset D in which n requirements samples *R*_1_, *R*_2_, .....*R*_*n*_ are annotated against a set of k unique labels *L* = λ_1_, λ_2_, ......., λ_*k*_. Dataset D comprises of K unique labels; therefore, it is transformed into k different datasets *D*_λ_*j*__, where λ_*j*_ = 1, .....*k*. Each *D*_λ_*j*__ dataset comprises of n requirements and each requirement is annotated against 1 or 0. Specifically, if requirement belongs to λ_*j*_ class, it is annotated as 1 otherwise 0.

In Figure 2, binary relevance method of data transformation module illustrates a hypothetical dataset having six requirements annotated against different combinations of four classes, namely security, availability, usability, and privacy. After transformation, four different datasets are formulated and each dataset represents to a unique class. The dataset that represents to privacy class contains 1 labels against the requirements which belongs to privacy class and it contains 0 labels for all other requirements that do not belong to privacy class. This approach deals each class independently and ignores label dependencies which misinterprets hidden correlations in data.

##### 3.1.2.2 Label powerset

This method transforms multi-label classification data into multiclass data, by considering each unique combination of labels as a separate class (Tsoumakas and Vlahavas, 2007). Transformed multiclass dataset may contain 2^|*L*|^ unique classes, where *L* denotes number of labels in original dataset. In Figure 2, LP method-based data transformation module illustrates a hypothetical multi-label dataset and after transformation generated multiclass dataset. It can be seen from Figure 2 six requirement samples belong to different combinations of four classes, namely, security, privacy, availability and usability. Requirement *R*_1_ belong to usability and security classes, Requirement *R*_2_ belongs to privacy and secuirty classes, and Requirement *R*_3_ belongs to security, privacy, and usability classes. Dataset contains four different combinations of classes, LP set assigns a unique integer to each unique combinations, and transformed data contain four unique labels.

Unlike BR, LP is competent in preserving labels co-relation by assigning unique labels to every distinct combination of labels. However, data transformed through LP become highly imbalance because it may contain up to 2^|*L*|^ labels. This results in varying numbers of requirements across different labels, with some labels having a significant number of requirements while others may have only a few requirements.

##### 3.1.2.3 Classifier chain

Similar to BR, CC also transforms multi-label dataset into L binary datasets, where L represents number of unique labels in original dataset. However unlike BR, CC builds chain of classifiers, and feature space of each binary classifier is extended with the labels predicted by the classifiers prior to itself in the chain. It considers label dependencies by using the order of labels to capture correlations.

To briefly understand CC-based data transformation, consider Figure 2 data transformation module Classifier Chain method illustrates four datasets that are formulated after transformation process. These datasets are formulated from multi-label data shown in Label powerset method. In transformed datasets, first data contain only requirements and their associated labels either o or 1. Second dataset input space contains requirements and output of previous dataset; similarly, third dataset input space contains requirements and outputs of previous both datasets.

##### 3.1.2.4 Classification module

After transforming multi-label classification data into binary or multiclass data, to perform requirements classification proposed framework facilitates nine different classifiers, namely, SVM (Tong and Koller, 2001), NB (Watkins, 1989), LR (LaValley, 2008), AdaBoost (Margineantu and Dietterich, 1997), RF (Breiman, 2001), DT (Quinlan, 1996), GB (Friedman, 2021), XGB (Chen et al., 2015), and ET (Geurts et al., 2006). SVM (Tong and Koller, 2001) makes use of kernel functions for transforming input feature space into more comprehensive feature space that is utilized for learning optimal hyperplane which discriminates requirements samples into predefined classes. NB (Watkins, 1989) treats all input features independently and iteratively computes probabilities of all classes with respect to input features of requirements. LR (LaValley, 2008) classifies data by finding the best-fit S-shaped curve that represents the probability of a binary outcome based on input features. DT recursively partitions data into subsets by selecting the most informative features at each step and creates a flowchart-base structure for classification (Quinlan, 1996). The ET enhances DT by introducing additional randomness in feature selection and threshold values for node splitting, resulting in a more diverse ensemble that mitigates overfitting (Geurts et al., 2006). RF (Breiman, 2001), AdaBoost (Margineantu and Dietterich, 1997), and GB (Friedman, 2021) are ensemble algorithms that combine multiple weak classifiers to create a powerful final classifier. XGB (Chen et al., 2015) is an advanced version of GB that uses tree-based models to create final classifier.

#### 3.1.3 Algorithm adaptation module

Algorithm adaptation methods are developed by adapting existing algorithms to more effectively align with multi-label data. These methods modify multiclass classification algorithms (KNN, NB, RF, LR, etc) to deal with multi-label classification. In this context, various classification algorithms, including Binary Relevance k-Nearest Neighbors (BRkNN; Spyromitros et al., 2008), Instance-Based Learning by Logistic Regression for Multi-Label learning (IBLRML; Cheng and Hüllermeier, 2009), Multi-Label k-Nearest Neighbors (MLkNN; Zhang and Zhou, 2007), RFBoost (Al-Salemi et al., 2016), MP-Boost (Esuli et al., 2006), Multi-Label Hierarchical ARAM Neural Network (MLARAM; Benites and Sapozhnikova, 2015), and Multi-label twin support vector machine (MLTSVM; Chen et al., 2016) are adapted from single-label classifiers, namely, kNN, SVM, and AdaBoost (Freund and Schapire, 1997). Algorithm adaptation module of proposed framework is enriched with four most widely used algorithm adaption techniques (MLkNN, *BRkNN*_*a*_, *BRkNN*_*b*_, and MLARAM), which are briefly summarized below.

##### 3.1.3.1 Multi-label k-nearest neighbors

MLkNN algorithm is an extension of standard KNN algorithm and utilizes maximum posterior principle to predict label set of an instance (Zhang and Zhou, 2007). This method predicts if an instance should be labeled with a specific label λ_*k*_ by analyzing whether a sufficient number of its k-nearest neighbors are also labeled with λ_*k*_.

For an unknown instance *R*_*i*_, it predicts if *R*_*i*_ should have label λ_*k*_ by comparing the probabilities: P(*R*_*i*_ having label λ_*k*_ | number of k-nearest neighbors labeled λ_*k*_) and P(*R*_*i*_ not having label λ_*k*_ | number of k-nearest neighbors labeled λ_*k*_). The objective of Bayes theorem is to compare P(*R*_*i*_ having label λ_*k*_) × P(number of k-nearest neighbors labeled λ_*k*_ | *R*_*i*_ having label λ_*k*_) with P(*R*_*i*_ not having label λ_*k*_) × P(number of k-nearest neighbors labeled λ_*k*_ | *R*_*i*_ not having label λ_*k*_), which can be computed from data.

##### 3.1.3.2 Binary relevance KNN

Binary Relevance KNN (BRkNN; Spyromitros et al., 2008) is another type of adaptation of kNN method. Instead of using binary transformation strategy with kNN algorithm in one-versus-all transformation manner, BRkNN (Spyromitros et al., 2008) expands KNN algorithm's functionality to allow separate predictions against each label. To avoid empty results for any test scenario, BRkNN (Spyromitros et al., 2008) uses percentage of predicted label's k-nearest neighbors to determine label confidence. Finally, BRkNN (Spyromitros et al., 2008) only assigns labels to specific instances if their confidence level exceeds a certain threshold.

##### 3.1.3.3 Multi-label hierarchical ARAM neural network

Multi-Label Hierarchical ARAM Neural Network (MLARAM) is powerful expansion of Adaptive Resonance Associative Map (ARAM), specifically designed to handle multidimensional data. This version introduces an additional ART layer that accelerates classification and supports development of large clusters of learned prototypes. Particularly, this concept is advantageous for multi-label text classification applications.

#### 3.1.4 Optimization module

Machine learning classifiers have a variety of trainable hyper-parameters, and utilization of optimal hyper-parameters (MacKay, 1996) can significantly boost their performance. Specifically, random forest classifier has two hyper-parameters: “number of estimators” and “maximum depth”. “Number of estimators” defines number of decision trees that classifier uses to predict class, and “maximum depth” denotes tree depth. Similarly, SVM classifier has two different hyper-parameters “c” and “gemma”, and “c” value represents regularization factor that makes trade-off between maximizing the margin between different classes and minimizing the classification error on the training data. Similarly, gemma value denotes shape of decision boundary. Furthermore, the hyper-parameter “learning rate (lr)” controls the step size of parameter updates during training of AdaBoost and GB classifiers.

The Bayesian model's performance can be enhanced by selecting the best values for the hyper-parameters: “*lamba*_1_,” “*lamba*_2_,” “*alpha*_1_,” “*alpha*_2_,” and “number of iterations (*n*_*it*_)” that calculates gamma distribution. In existing requirements classification predictors, best values of hyper-parameters are found through extensive manual experimentation or grid search-based strategy (Liashchynskyi and Liashchynskyi, 2019). However, in large computational frameworks while performing experimentation over diverse types of datasets with multiple predictive pipelines, it is difficult to find optimal hyper-parameters of multiple classifiers through manual experimentation. Similarly, grid search-based experimentation with wide hyper-parameter search space requires a lot of computational power and time.

The proposed computational framework contains four different representation learning strategies, three data transformation methods, and nine machine learning classifiers. Therefor, using these two modules, there is possibility of developing 4 × 3 × 9 = 108 predictive pipelines. Similarly, four different representation strategies along with four algorithm adaption strategies makes 4 × 4 = 16 predictive pipelines. To perform performance comparison, of all 108 + 16 = 124 predictive pipelines over 3 requirements datasets require to perform 124 × 3 = 372 experiments with classifier's default hyper-parameters. A fair performance comparison of all predictive pipelines requires each predictive pipeline with best hyper-parameters; to accomplish this task, we utilize particle swarm optimization (PSO) that finds optimal subset of hyper-parameters using smart strategy (MacKay, 1996).

Considering the need of an automated method competent in finding optimal hyper-parameters of machine learning methods, Kennedy and Eberhart (1995) proposed PSO optimizer that makes use of two different natural behaviors, namely, birds swarming behavior and biological processes optimization strategy to find optimal subset of hyper-parameter.

- **Swarming behavior:** The study of collective behavior observed in groups of animals and flocks of birds. These groups exhibit coordinated movement and interaction, leading to emergent behavior at the group level.
- **Evolutionary computing:** The domain of genetic algorithms that utilizes principles of biological evolution to find optimal solutions for complex problems.

**Figure 3:**
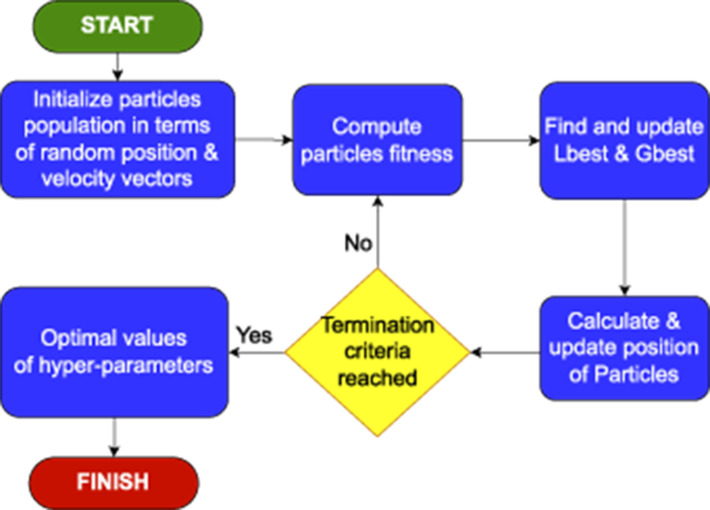
Graphical illustration of complete workflow of particle swarm optimizer.

The working of PSO optimizer can be summarized in five different steps, which are graphically illustrated in Figure 3. (1) First of all, it generates a population of particles within a predefined search space, where each particle represents potential solution. It randomly initializes position and velocity of each particle. (2) Then, it evaluates fitness of each particle based on particle's performance against an objective function. (3) Afterward, it updates local best (Lbest) and global best (Gbest) positions based on evaluation of each particle's fitness. (4) Then, it adjusts velocity of each particle based on its current velocity and tendency for finding Lbest and Gbest values, respectively. Equation 6 demonstrates mathematical formulation for updating a particle's velocity.


(6) $$\begin{array}{l} v_{i}^{\left(t+1\right)}=wv_{i}^{\left(t\right)}+c_{1}r_{1}\left[{\hat{x}}_{i}^{\left(t\right)}-x_{i}^{\left(t\right)}\right]+c_{2}r_{2}\left[g^{\left(t\right)}-x_{i}^{\left(t\right)}\right] \end{array}$$

In Equation 6, $v_{i}^{\left(t\right)}$ and $v_{i}^{\left(t+1\right)}$ represent current and new velocity of *i*^*th*^ particle at *t*^*th*^ and (*t*+1)^*th*^, respectively. *w* is a constant that controls effect current velocity $v_{i}^{\left(t\right)}$ on new velocity for *i*^*th*^ particle. *c*_1_ and *c*_2_ denote tendency of particle to move toward pBest and gBest, respectively. *r*_1_ and *r*_2_ are random values that range between 0 and 1, while $x_{i}^{\left(t\right)}$ denotes current position of *i*^*th*^ particle at *t*^*th*^ iteration.

It utilizes new velocity to update particle's position in search space, as illustrated in Equation 7.


(7) $$\begin{array}{l} x_{i}^{\left(t+1\right)}=x_{i}^{\left(t\right)}+v_{i}^{\left(t+1\right)} \end{array}$$

(5) It iteratively repeats aforementioned steps (2–4) until it reaches optimal solution.

In a nutshell, the particles move in predefined search space by utilizing velocity and position of particles to find Lbest and Gbest values though iterative process. Finally, the particles in search space converge at optimal solution.

#### 3.1.5 Adapted deep learning predictors

Apart from requirements multi-label classification, for other different types of multi-label text classification such as biomedical question classification (Wasim et al., 2019; Sarrouti et al., 2015), hate speech detection (Ibrohim and Budi, 2019; Ameur and Aliane, 2021), tag recommender (Lei et al., 2020), and medical codes and categories assignment (Pakhomov et al., 2006; Samanta, 2021), researchers have proposed several deep learning predictors that are competent in accurately categorizing textual samples into relevant classes (Mohammed and Kora, 2022; Dogra et al., 2022; Rasool et al., 2022). Researchers have leveraged diverse types of deep learning architectures for the development of multi-label text classification predictors including CNN (Liu et al., 2017; Xu et al., 2017; Peng et al., 2019), RNN (Du et al., 2019; Liu et al., 2016; You et al., 2018), hybrid networks (CNN + RNN; Lai et al., 2015), CNN with attention mechanism (Gargiulo et al., 2018; Kurata et al., 2016; Peng et al., 2018; Shimura et al., 2018), and regional embeddings-based predictors (Bojanowski et al., 2017; Qiao et al., 2018; Akbik et al., 2018).

Within this array of predictors, each type of predictor possesses its unique set of strengths and weaknesses. Existing research findings demonstrate that RNN-based predictors successfully capture long distance dependencies between features but extract less discriminative feature (Du et al., 2019; Liu et al., 2016; You et al., 2018), while CNN-based predictors efficiently extract discriminative features but remains fail in acquiring long range dependencies among features (Yin et al., 2017). CNN- and RNN-based approaches surpass each other performance, depending on characteristics of corpus features. Hybrid predictors reap the benefits of both CNN and RNN architectures and produce better performance in comparison with standalone CNN- or RNN-based predictors (Lai et al., 2015). Attention-based CNN or RNN yields more informative features because attention preserves interdependence of sentences in a document (Yang et al., 2016).

With an aim to explore the potential of various CNN, RNN, attention-based, and hybrid predictors for the task of requirements multi-label classification, we adapted nine diverse types of deep learning predictors as baseline methods. The original manuscripts of these predictors have provided concise descriptions. Hence, in this context, we have succinctly summarized their architectural intricacies shown in Table 2. Furthermore, a high level overview of these predictors is provided in Section 1 of Supplementary material.

**Table 2:** Comprehensive summary of adapted deep learning predictors.

**Predictor**	**CNN layers**	**RNN layers**	**Attention layers**	**Pooling layers**	**Dense layers**
Region embedding (Qiao et al., 2018)	-	-	-	-	1
FastText (Joulin et al., 2016)	-	-	-	-	1
TextCNN (Kim, 2014)	4	-	-	-	2
DPCNN (Liu and Liu, 2017)	6	-	-	2	1
TextVDCNN (Conneau et al., 2016)	9	-	-	1	3
AttentionConvNet (Yin and Schütze, 2018)	5	-	1	1	3
TextRNN (Liu et al., 2016)	-	1	1	-	1
DRNN (Wang, 2018)	-	2	-	-	2
TextRCNN (Lai et al., 2015)	3	1	-	1	1

### 3.2 Benchmark datasets

The proposed framework is evaluated on three public benchmark datasets: PROMISE, EHR-binary, and EHR-multiclass. Existing requirement classification predictors (AlDhafer et al., 2022; Slankas and Williams, 2013) performance is evaluated on these datasets. This enables proposed framework performance comparison with existing requirements classification predictors (AlDhafer et al., 2022; Slankas and Williams, 2013). A brief summary of these datasets is provided below.

#### 3.2.1 Promise

**Figure 4:**
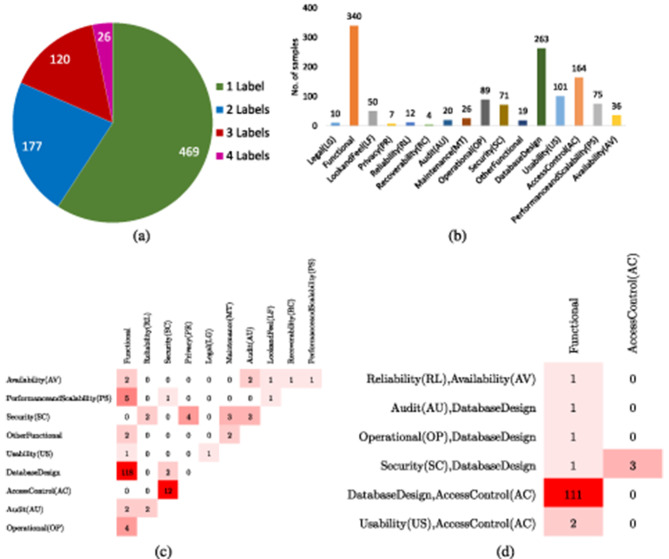
**(a)** Descriptive statistic of PROMISE multi-label dataset segregation of instances in terms of label cardinality. **(b)** Count of instances in each class. **(c)** Dense Bi-Label Confusion Matrix. **(d)** Dense Tri-Label Confusion Matrix.

Slankas and Williams (2013) presented PROMISE dataset that contains 792 requirements annotated against 15 different classes. Table 3 depicts few samples of promise ML dataset along with class labels. Overall, based on sample-to-label distribution, 792 requirements samples can be categorized into 4 different categories such as samples that belong to only 1 class at a time, samples that belong to 2 classes at a time, similarly, samples that belong to 3 and 4 classes at a same time. Figure 4a graphically illustrates distribution of samples into 1, 2, 3, and 4 labels, where it can be seen that most of the samples belong to only one label and with the increase in number of labels distribution of samples is reduced. Figure 4 illustrates overall sample-to-label distribution into four different categories, while Figure 4b more briefly describes how 469 samples are distributed into each class. Similarly, Figure 4c illustrates 177 samples that belong to two different classes are how distributed into different pairs of classes. Similarly, Figure 4d briefly describes distribution of samples into three different labels. Hence, imbalanced label distribution of promise dataset poses significant challenges, specifically, fewer samples exist for requirements with multiple labels which might lead to poor predictive performance for requirements belonging to multiple classes.

**Table 3:** Samples of promise dataset with class labels.

**Sample**	**Labels**
All credit card information will be encrypted in the database	Maintenance, Security
The system shall protect private information in accordance with the organization's information policy	Security, Privacy
All customer information will be stored on a secure database accessible only to authorized personnel	Maintenance, Security, Access Control
Daily usage statistics should be logged and accessible by the administrator	Functional, Audit, Database Design, Access Control

#### 3.2.2 Electronic health records

Slankas and Williams (2013) collected 5,722 requirements from 12 different healthcare domain projects and manually annotated these requirements in two different ways. The authors presented Electronic Health Records (EHR) dataset of two different versions, namely, binary multi-label classification and multiclass multi-label classification. In binary multi-label classification version of EHR dataset, 5,722 requirements are annotated against three different classes, namely, functional, non-functional, and both. Specifically, in this version of dataset, 2758 samples belong to functional class, 1889 samples belong to non-functional class, and 1075 samples belong to both functional and non-functional classes.

**Figure 5:**
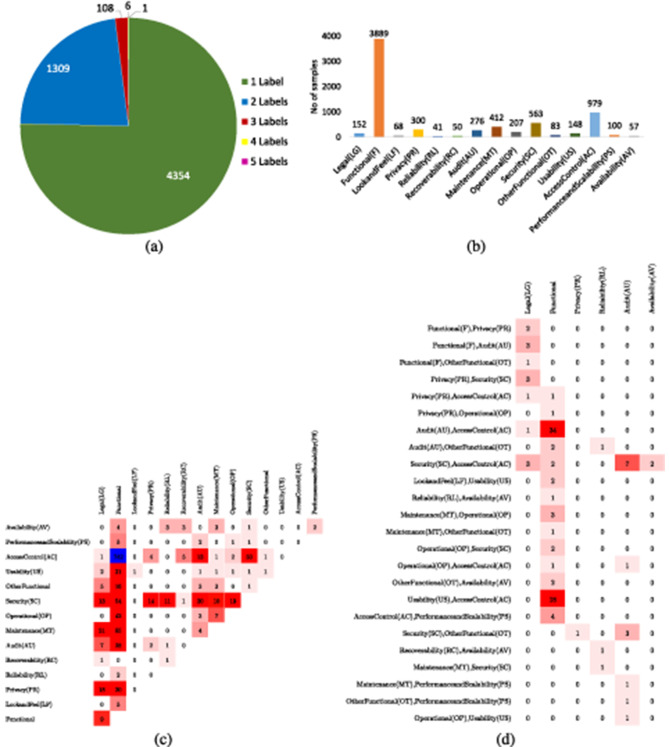
**(a)** Descriptive statistic of EHR-Multiclass dataset with segregation of instances in terms of label cardinality. **(b)** Count of instances in each class. **(c)** Dense Bi-Label Confusion Matrix. **(d)** Dense Tri-label Confusion Matrix.

In multiclass multi-label classification version of EHR dataset, 5,722 requirements are annotated against 15 different classes, namely, legal, functional, look and feel, privacy, reliability, recoverability, audit, maintenance, operational, security, usability, access control, performance and scaleability, availability, and other functional. Figure 5a graphically illustrates the distribution of multiclass multi-label dataset across multiple labels, that is, samples belonging to one class, two classes, three classes, four classes, and five classes. It is evident from Figure 5a that a few requirements samples belong to 3, 4, and 5 labels. Figure 5b represents label-wise distribution among uni-label requirement samples. Similarly, Figures 5c, d represent the distribution of 1309 and 108 samples containing bi- and tri-labels, respectively. Similar to the PROMISE dataset, the EHR dataset also has a limited number of requirement samples with multiple labels, which poses challenges for effective multi-label classification and may affect model performance on such instances.

### 3.3 Evaluation measures

Primarily in binary or multiclass classification, a requirement sample belongs to one class at a time; hence, prediction can be entirely correct or incorrect. Contrarily, in multi-label classification, a requirement sample belongs to more than one class at the same time; hence, predictions can be entirely correct, entirely incorrect, partially correct, or incorrect. Therefore, evaluation of multi-label classification predictors is more difficult than evaluation of binary or multiclass classification predictors (El Kafrawy et al., 2015). Multi-label classification-based evaluation measures are categorized into two groups: example-based (Giraldo-Forero et al., 2015) and label-based (El Kafrawy et al., 2015). It is considered that evaluation measures that fall under the category of label-based are incapable of considering label interdependence; hence, the majority of multi-label classification techniques have been evaluated through example-based measures (Giraldo-Forero et al., 2015).

To facilitate large-scale performance evaluation criteria, proposed framework is enriched with eight distinct evaluation measures, namely, accuracy, precision, recall, f1-score, average precision, coverage, ranking loss, hamming loss, and one-error. Accuracy (Alshanqiti and Namoun, 2020) computes ratio of correctly predicted labels to actual number of labels. Precision (Jiang et al., 2014) calculates the total number of correctly predicted instances among all instances. Recall (Pereira et al., 2018) estimates the number of times a specific label is accurately predicted. F1-score (Bénédict et al., 2021) is harmonic mean of recall and precision. A higher value for these metrics indicates better performance of the classifier. Average precision calculates average of all precision at different recall levels to assess varying class relevance.

Coverage (Hüllermeier et al., 2020) quantifies proportion of instances that have at least one relevant class correctly predicted among all instances. It indicates the predictor's ability to capture the full set of relevant classes for a given dataset. Ranking loss (Zhang and Zhou, 2005) quantifies the number of times the incorrect label appears prior to actual label. Lower numbers of these ranking measures indicate more powerful classifier performance. The Hamming loss (Dembczyński et al., 2012) calculates the likelihood of inaccurately classifying a label combination by focusing on labels that are either wrongly predicted (prediction error) or un-predicted (missing error, where the prediction of a relevant label is absent).


(8) $$f(x)=\left\{ \begin{array}{l} \;Accuracy=\frac{1}{N}\sum_{i=1}^{N}\left[\frac{a_{i}\wedge p_{i}}{a_{i}\vee p_{i}}\right]\\ \;Precision=\frac{1}{N}\sum_{i=1}^{N}\left[\frac{a_{i}\wedge p_{i}}{p_{i}}\right]\\ \;Recall=\frac{1}{N}\sum_{i=1}^{N}\left[\frac{a_{i}\wedge p_{i}}{a_{i}}\right]\\ Average=\frac{1}{M}\sum_{i=1}^{N}\sum_{y\varepsilon Y_{i}}\frac{y^{\prime }f_{rank}(x_{i},y^{\prime })\leq f_{rank}(x_{i},y),y^{\prime }\epsilon Y_{i}}{f_{rank}(x_{i},y)}\\ \;F1-score=\frac{1}{N}\sum_{i=1}^{N}\left[\frac{2*(Pre(n_{i})*Rec(n_{i})}{Pre(n_{i})+Rec(n_{i})}\right]\\ \;Coverage=\frac{1}{N}*\sum_{o=1}^{N-1}max_{j:\;a_{ij}=1}rank_{ij}\\ \;RankingLoss=\frac{1}{N}*\sum_{o=1}^{N-1}\frac{1}{a_{i}*(n_{labels}-a_{i})}\\ \;HammingLoss=\frac{1}{N}\sum_{i=1}^{N}\sum_{j=1}^{Len}\left[I(a_{i}^{j}\neq b_{i}^{j})\right] \end{array}\right.$$

In Equation 8, *N* represents total number of samples, *n*_*i*_ denotes *i*^*th*^ sample out of *n* samples, *a*_*i*_ denotes actual class label, *p*_*i*_ represents predicted label of *n*_*i*_ sample, *Len* stands for length of sample, *j*^*th*^ stands for class index, and ∨ and ∧ stand for logical OR and AND operator, respectively.

## 4 Experimental setup

The proposed framework is developed on top of eight APIs, namely, scikit-learn,^2^ numpy,^3^ math,^4^ scipy,^5^ pandas,^6^ matplotlib,^7^ FastAI,^8^ and pytorch.^9^ Following experimental criteria of existing studies (AlDhafer et al., 2022; Slankas and Williams, 2013), we perform experimentation in 10-fold cross-validation setting. In this this setting iteratively, 1-fold is taken as test set and other 9-fold are used for model training. Specifically, in EHR dataset among 5722 samples, 572 samples are used as test set and remaining 5150 samples are used for training. Furthermore, all predictive pipeline performance is computed using both micro and macro versions of precision, recall, and F1-measures. To investigate impact of hyper-parameters on classifier performance, We conducted experimentation under two distinct settings. In the first setting, we evaluated classifiers with their default hyper-parameters. In the second setting, we choose a suitable hyper-parameter space as shown in Table 4 and used PSO to discover the optimum hyper-parameters within the chosen space. Furthermore, for adapted deep learning predictors, we have utilized binary cross entropy loss with logits (Ruby and Yendapalli, 2020) along with Adam optimizer (Zhang, 2018).

**Table 4:** Search space for different problem transformation and algorithm adaption classifiers.

**Problem transformation classifier**	**PSO search space**	**Algorithm adaptation**	**PSO search space**
Adaptive Boosting (AB)	learning rate (0.00001,..,1) n_estimators (10,...,1000)	Binary Relevance k-Nearest Neighbors (*BRkNN*_*a*_)	k (1,..,10)
Gradient Boost(GB)	learning rate (0.00001,...,1) max_depth (10,..,1000) min samples leaf (5,...,100) min samples split (10,...,1000) n_estimators (10,...,1000)	Binary Relevance k-Nearest Neighbors (*BRkNN*_*b*_)	k (1,..,10)
Support Vector Classifier (SVC)	C (1,..,1000) degree (1,...,4) gamma (0.000001,...,1) max iterations (500,...,1000) tol (0.00001,...,0.0001)	Multi-LabelHierarchical ARAMNeural Network(MLARAM)	Threshold Vigilance (0.01,...,1)
Logistic Regression (LR)	C (1,..,1000) max iterations (500,...,1000) tol (0.00001,...,0.0001)	Multi-Label k-Nearest Neighbors (MLKNN)	k (1,..,10)
Extreme Gradient Boosting (XGB)	learning rate (0.00001,..,1) max-depth (10,...,1000) n_estimators (10,...,1000)		
Decision Tree (DT), Random Forest (RF), Extra Tree (ET)	max_depth (10,...,1000) min sample leaf (5,...,100) min sample split (10,...,1000) n_estimators (10,...,1000)		

## 5 Results

This section performs an extensive examination of proposed MLR framework predictive pipelines performance across three benchmark datasets. First of all, it shows swarm optimizer-based hyper-parameters optimization impact on data transformation and algorithm adaptation-based predictive pipeline performance. Second, it investigates data transformation and algorithm adaptation-based predictive pipeline performance along with four different representation learning strategies. Third, it analyze performance of nine adapted deep learning predictors along with three different word embeddings. Fourth, it performs performance comparison of top-performing machine and deep learning predictors. Finally, it compares MLR framework top-performing predictive pipeline performance with state-of-the-art multi-label requirements classification predictor performance. Finally, it performs a case study in which proposed predictor performance is compared with BERT language model for software reviews multi-label classification task.

As MLR framework produces multiple predictive pipelines, therefore to facilitate readers we named data transformation-based predictive pipelines as SR-DT-CL and algorithm adaptation-based predictive pipelines as SR-AACL. In SR-DT-CL-based pipelines, SR can be any method from four representation learning methods namely TFIDF, OkapiBM25, word2vec, and FastText. Similarly, DT can be any method from data transformation-based three methods BR, LP, and CC and CL can be any classifier from nine classifiers. Specifically, OkapiBM25 representation learning method, BR data transformation method, and RF classifier predictive pipeline are named as OkapiBM25-BR-RF. Similarly, OkapiBM25 representation-based predictive pipeline with MLNN algorithm adaption technique is named as OkapiBM25-MLKNN. Furthermore, EHR-Binary and EHR-Multiclass datasets are named as EHR-B and EHR-M, respectively.

### 5.1 Performance analysis of requirements multi-label classification pipelines using default and optimized hyper-parameters

This section provides a comprehensive summary about how swarm optimizer affects the performance of multi-label classification predictors. To investigate impact of hyper-parameters on predictors performance, over three benchmark datasets (promise, EHR-B, EHR-M), we performed experimentation under two distinct experimental settings. In the first setting, data transformation and algorithm adaptation-based predictive pipelines are evaluated using default hyper-parameters of OkapiBM25 and classifiers. In the second setting, first for each dataset by using 70% data, we employed swarm optimizer to find optimal set of OkapiBM25 and classifiers hyper-parameters from a wide space of hyper-parameters shown in Table 4. Furthermore, using optimal hyper-parameters, we performed experimentation using all predictive pipelines.

Table 5 illustrates predictive pipelines (OkapiBM25-DT-CL, OkapiBM25-AACL ) performance across three benchmark datasets using default and optimized hyper-parameters. In Table 5, the Δ column illustrates performance gain achieved by predictive pipelines with optimized hyper-parameters in comparison with their performance with default hyper-parameters. A bird's-eye view of Δ column illustrates that all predictive pipelines achieved performance gains with optimized hyper-parameters across all three datasets. However, a closer examination of Table 5 reveals that some predictive pipelines achieved significant performance gain, while others only experienced marginal improvements in performance. This performance distinction primarily arises due to the fact that the performance of certain classifiers is heavily dependant on hyper-parameters, whereas for other classifiers, hyper-parameters have a relatively minor impact on their performance. Specifically, eight predictive pipelines, namely, OkapiBM25-DT-AB, OkapiBM25-DT-LR, OkapiBM25-DT-RF, OkapiBM25-DT-SVC, OkapiBM25-DT-GB, OkapiBM25-DT-ET, OkapiBM25-*BRkNN*_*a*_, and OkapiBM25-MLARAM, showed higher performance gain with optimized hyper-parameters. After observing performance improvements achieved by various predictive pipelines, the answer to research question three becomes evident: incorporating swarm optimization in predictive pipelines proves to be beneficial.

**Table 5:** Performance analysis of data transformation and algorithm adaptation-based requirements multi-label classification predictive pipelines using OkapiBM25 representation method and classifiers default and swarm optimizer-based optimal hyper-parameters.

**Data transformation approaches**	**Classifier**	**Promise**			**EHR-Binary**			**EHR-Multiclass**		
		**OkapiBM25 default**	**OkapiBm25 optimized**	Δ	**OkapiBM25 default**	**OkapiBm25 optimized**	Δ	**OkapiBM25 default**	**OkapiBm25 optimized**	Δ
Binary relevance	AB	0.499	0.577	0.078	0.822	0.855	0.033	0.711	0.728	0.017
	DT	0.520	0.535	0.016	0.785	0.798	0.013	0.727	0.733	0.006
	ET	0.468	0.477	0.008	0.872	0.882	0.010	0.733	0.747	0.015
	GB	0.468	0.553	0.085	0.848	0.896	0.048	0.739	0.781	0.042
	LR	0.601	0.648	0.048	0.893	0.894	0.001	0.785	0.793	0.008
	NB	0.298	0.3025	0.005	0.854	0.874	0.021	0.664	0.677	0.013
	RF	0.358	0.400	0.042	0.858	0.896	0.038	0.700	0.715	0.015
	SVC	0.487	0.508	0.021	0.729	0.895	0.166	0.606	0.749	0.143
	XGB	0.418	0.431	0.012	0.874	0.886	0.011	0.748	0.761	0.013
Label powerset	AB	0.388	0.434	0.046	0.782	0.834	0.052	0.601	0.606	0.004
	DT	0.462	0.474	0.012	0.810	0.824	0.014	0.722	0.722	0.000
	ET	0.616	0.666	0.050	0.874	0.896	0.022	0.770	0.787	0.017
	GB	0.606	0.627	0.020	0.845	0.894	0.049	0.742	0.751	0.010
	LR	0.750	0.754	0.004	0.892	0.905	0.013	0.814	0.834	0.020
	NB	0.498	0.517	0.019	0.862	0.874	0.013	0.658	0.682	0.024
	RF	0.541	0.627	0.086	0.849	0.880	0.031	0.675	0.758	0.083
	SVC	0.354	0.672	0.319	0.607	0.892	0.285	0.606	0.824	0.218
	XGB	0.534	0.570	0.036	0.880	0.891	0.012	0.767	0.781	0.014
Classifier chain	AB	0.495	0.595	0.100	0.808	0.868	0.060	0.715	0.773	0.058
	DT	0.517	0.531	0.014	0.823	0.828	0.004	0.710	0.715	0.005
	ET	0.455	0.481	0.026	0.882	0.892	0.010	0.734	0.760	0.025
	GB	0.488	0.522	0.034	0.808	0.888	0.080	0.743	0.785	0.042
	LR	0.632	0.659	0.027	0.871	0.888	0.017	0.802	0.804	0.002
	NB	0.312	0.323	0.011	0.853	0.874	0.021	0.654	0.683	0.029
	RF	0.355	0.390	0.036	0.859	0.883	0.024	0.703	0.729	0.027
	SVC	0.523	0.534	0.011	0.730	0.896	0.165	0.606	0.807	0.201
	XGB	0.436	0.447	0.011	0.871	0.880	0.009	0.764	0.768	0.003
Algorithm adaptation	*BRkNN*_*a*_	0.378	0.437	0.060	0.778	0.802	0.025	0.589	0.761	0.172
	*BRkNN*_*b*_	0.091	0.092	0.001	0.783	0.824	0.042	0.010	0.013	0.002
	MLARAM	0.428	0.438	0.010	0.560	0.746	0.186	0.617	0.734	0.117
	MLKNN	0.491	0.494	0.003	0.811	0.829	0.018	0.690	0.763	0.073

### 5.2 Performance comparison of diverse types of text representation approaches along with data transformation and algorithm adaptation-based predictive pipelines

To find answers of four research questions (*Q*_1_, *Q*_2_, *Q*_3_, *Q*_4_) mentioned in Section 1, this section performs a comprehensive performance analysis of four representation learning approaches along with data transformation and algorithm adaptation-based predictive pipelines. Table 6 illustrates performance values of 27 data transformation and 4 algorithm adaptation-based predictive pipelines along with four different representation learning methods across 3 benchmark datasets. Furthermore, Supplementary Tables 1–12 illustrates in-depth performance analysis of 27 data transformation and 4 algorithm adaptation-based predictive pipelines based on 14 different evaluation measures using 4 different representation learning methods across 3 benchmark datasets, respectively.

**Table 6:** Performance comparison of data transformation and algorithm adaptation-based predictive pipelines using F1-score across three benchmark datasets.

**DT approach**	**Classifier**	**Promise**				**EHR-Binary**				**EHR-MultiClass**			
		**TFIDF**	**OkapiBM25**	**Word2vec**	**FastText**	**TFIDF**	**OkapiBM25**	**Word2vec**	**FastText**	**TFIDF**	**OkapiBM25**	**Word2vec**	**FastText**
Binary relevance	AB	0.507	0.577	0.437	0.446	0.819	0.855	0.780	0.807	0.710	0.728	0.659	0.667
	DT	0.547	0.535	0.349	0.35	0.808	0.798	0.689	0.703	0.727	0.733	0.560	0.571
	ET	0.484	0.477	0.250	0.277	0.873	0.882	0.813	0.862	0.728	0.747	0.626	0.650
	GB	0.487	0.553	0.377	0.399	0.852	**0.896**	0.843	0.853	0.744	0.781	0.677	0.679
	LR	0.298	**0.648**	0.048	0.246	0.895	0.894	0.820	0.849	0.681	**0.793**	0.589	0.653
	NB	0.277	0.302	0.395	0.482	0.892	0.874	0.712	0.762	0.641	0.677	0.568	0.605
	RF	0.394	0.400	0.273	0.284	0.853	0.896	0.811	0.859	0.698	0.715	0.643	0.627
	SVC	0.494	0.508	0.229	0.366	0.729	0.895	0.855	0.878	0.606	0.749	0.633	0.699
	XGB	0.442	0.431	0.412	0.444	0.877	0.886	0.860	0.874	0.749	0.761	0.716	0.715
Label powerset	AB	0.388	0.434	0.331	0.355	0.779	0.834	0.783	0.795	0.611	0.606	0.604	0.605
	DT	0.474	0.474	0.322	0.352	0.833	0.824	0.739	0.747	0.723	0.722	0.560	0.568
	ET	0.635	0.666	0.589	0.591	0.875	0.896	0.839	0.846	0.777	0.787	0.688	0.699
	GB	0.603	0.627	0.463	0.492	0.844	0.894	0.837	0.849	0.736	0.751	0.689	0.679
	LR	0.640	**0.754**	0.416	0.555	0.887	**0.905**	0.802	0.846	0.747	**0.834**	0.624	0.729
	NB	0.583	0.517	0.453	0.541	0.868	0.874	0.754	0.787	0.652	0.682	0.612	0.641
	RF	0.560	0.627	0.579	0.581	0.850	0.880	0.837	0.8454	0.750	0.758	0.689	0.699
	SVC	0.354	0.672	0.501	0.622	0.607	0.892	0.837	0.871	0.606	0.824	0.695	0.763
	XGB	0.551	0.570	0.560	0.605	0.878	0.891	0.865	0.874	0.762	0.781	0.714	0.729
Classifier chain	AB	0.503	0.595	0.456	0.482	0.812	0.868	0.790	0.815	0.718	0.773	0.672	0.674
	DT	0.535	0.531	0.337	0.346	0.829	0.828	0.730	0.7492	0.727	0.715	0.566	0.576
	ET	0.492	0.481	0.299	0.325	0.880	0.892	0.826	0.838	0.742	0.760	0.665	0.667
	GB	0.509	0.522	0.395	0.432	0.805	0.888	0.820	0.841	0.743	0.785	0.691	0.690
	LR	0.398	**0.659**	0.087	0.367	0.881	**0.888**	0.728	0.835	0.742	0.804	0.628	0.697
	NB	0.319	0.323	0.419	0.483	0.872	0.874	0.739	0.777	0.649	0.683	0.577	0.610
	RF	0.391	0.390	0.282	0.302	0.863	0.883	0.841	0.855	0.707	0.729	0.656	0.655
	SVC	0.49.5	0.534	0.329	0.522	0.729	0.896	0.816	0.863	0.606	**0.807**	0.677	0.735
	XGB	0.460	0.447	0.447	0.433	0.868	0.880	0.855	0.866	0.768	0.768	0.726	0.728
Algorithm adaptation	*BRkNN*_*a*_	0.457	0.437	0.082	0.342	0.813	0.802	0.572	0.809	0.741	0.761	0.421	0.678
	*BRkNN*_*b*_	0.089	0.092	0.075	0.086	0.819	0.824	0.572	0.808	0.011	0.013	0.008	0.009
	MLARAM	0.354	0.438	0.349	0.349	0.607	0.746	0.607	0.607	0.606	0.734	0.522	0.522
	MLKNN	0.456	**0.494**	0.013	0.448	0.814	**0.829**	0.695	0.819	0.761	**0.763**	0.602	0.723

Bold values indicate highest performance figure across all methods for all 3 datasets.

It is evident from Table 6 that all 31 (27 SR-DT-CL, 4 SR-AACL) predictive pipelines produce significantly better performance with TFIDF and OkapiBM25 representation learning methods as compared to their performance with pre-trained word embeddings methods. Word embeddings are regarded as superior representation learning techniques in comparison with traditional bag of word-based methods. However, their utility in requirements classification encounters limitations due to the scarcity of available data. Developing comprehensive word embeddings from a small requirements data seems impractical. Although different types of pre-trained word embeddings are publicly available, they are typically constructed from generic corpora such as English Wikipedia and English CoNLL17 corpus (Kutuzov et al., 2017). Unfortunately, these pre-trained embeddings lack many keywords specific to requirements, ultimately influencing the performance of predictors.

In Table 6, experimental results reveal that among both pre-trained word embeddings across three benchmark datasets, all 31 predictive pipelines produce better performance with FastText pre-trained word embeddings. In comparison with word2vec, FastText pre-tranied embeddings aids classifiers to produce better performance because FastText generates pre-trained word embeddings by extracting subwords information that also helps to acquire out of vocabulary words embeddings. On the other hand, among two statistical representation learning approaches, OkapiBM25 encoder outperforms TFIDF based representation across all three benchmark datasets. Particularly, OkapiBM25 transforms requirements into statistical vectors by efficiently handling requirements length variability effect on words term frequencies. Moreover, it also assigns less scores to most frequent terms.

Specifically, among all FastText pre-trained word embeddings-based predictive pipelines, FastText-LP-ET, FastText-BR-RF, and FastText-LP-SVC achieve best performance over promise, EHR-B, and EHR-M, respectively. Among TFIDF representation-based predictive pipelines, TFIDF-LP-ET demonstrates superior performance over promise and EHR-M datasets, while TFIDF-BR-LR produces highest performance over EHR-B dataset. Contrarily, among all OkapiBM25 representation method-based predictive pipelines, OkapiBM25-LP-LR produces highest performance across all three datasets. Furthermore, OkapiBM25-LP-LR beats performance of all other SR-DT-CL and SR-AACL predictive pipelines with significant performance margin. In a nutshell, TFIDF and pre-trained word embeddings lack to generate comprehensive statistical representation of requirement for accurate class predictions. Furthermore, these representation learning methods also remain fail to provide a generic predictive pipeline across all three datasets.

Aforementioned performance analysis demonstrates that, as an answer to research question 1, the combination of the label powerset data transformation approach and the logistic regression classifier forms the most effective predictive pipelines. Regarding research question 2, the OkapiBM25 method showcases a more comprehensive representation when compared to TFIDF and pre-trained word embeddings. Moreover, addressing research question 3, it is evident that the development of a generic requirements multi-label classification predictive pipeline is feasible as OkapiBM25-LP-LR outperformed all other predictive pipelines across all the datasets.

### 5.3 Performance analysis of adapted deep learning predictors

**Figure 6:**
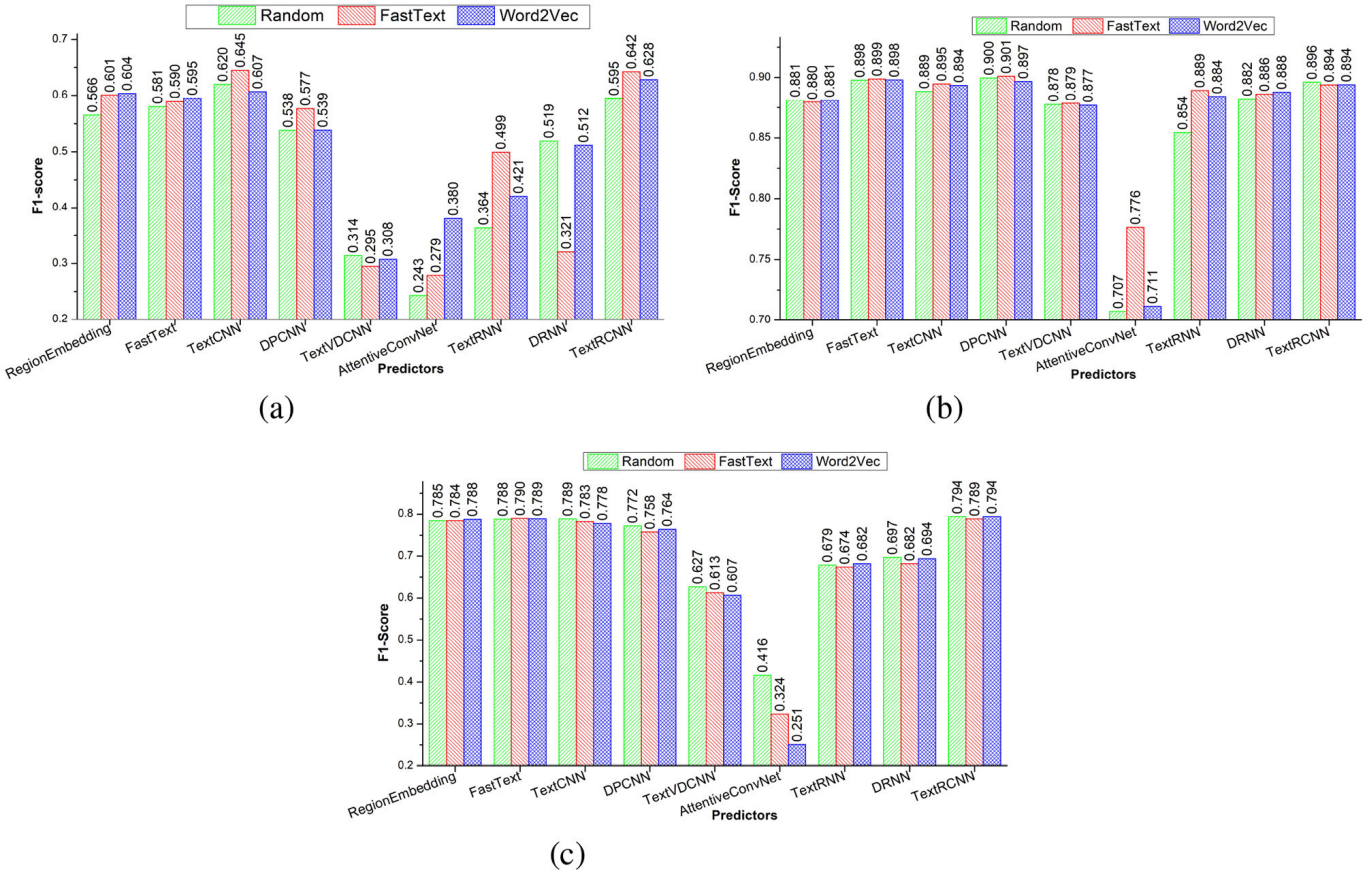
Performance comparison of adapted deep learning predictors based on different embeddings using three public benchmark dataset. **(a)** Promise. **(b)** EHR-Binary. **(c)** EHR-Multiclass.

To address research question 5, this section performs an analysis of how three distinct word embedding methods influence predictive performance of nine adapted deep learning predictors. Figures 6a–c graphically illustrate three distinct word embeddings (Random, Word2vec, FastText) impact on nine deep learning predictors F1-scores across three benchmark datasets, namely, promise, EHR-B, and EHR-M, respectively. Furthermore, Supplementary Tables 13–15 illustrates in-depth performance analysis nine adapted deep learning predictors using 14 evaluation measures over promise, EHR-B, and EHR-M datasets, respectively.

It can be seen in Figure 6a over promise dataset, two predictors, namely, AttentionConvNet and TextRNN, produce better performance with word2vec embeddings as compared to their performance with other two embeddings (random, FastText). Similarly, FastText embddings assisted three predictors (TextCNN, DPCNN, TextRCNN) to produce better performance in comparison with other two embedding methods (random, word2vec). Two predictors namely region embedding and FastText produce better and similar performance with word2vec and FastText embeddings as compared to their performance on random embeddings. Random embeddings assisted two predictors (TextVDCNN, DRNN) to perform better as compared to two pre-trained embeddings. Similarly, it can be seen in Figures 6b, c, across two datasets EHR-B and EHR-M, seven predictors, namely, FastText, Region embedding, TextCNN, DPCNN, TextVDCNN, DRNN, and Text RCNN, produce almost similar performance for three distinct types of embeddings. Although TextRNN produces better performance with FastText based embeddings over EHR-B dataset, it produce similar performance with all three embeddings over EHR-M dataset. AttentionConvNet predictor produces better performance with FastText embeddings over EHR-B but in case of EHR-M dataset it produces better performance with random embeddings.

Overall, it can be concluded across EHR-B and EHR-M datasets, pre-trained embeddings remain fail to enhance the performance of the predictors. Pre-trained word embeddings remain fail to enhance the performance of predictors because these embeddings have been developed on generic data and lack most of the requirements key words. On the other hand, although pre-trained embeddings lacks many requirements key words but still over promise dataset predictors produce better performance with pretained embeddings. Reason behind this performance distinction is small size of promise data as compared to EHR-B and EHR-M dataset.

It is evident from Figure 6, among three CNN-based predictors, TextCNN and DPCNN predictors produce almost similar and better performance, while VDCNN predictor remain least performer. The poor performance of VDCNN predictor is due to its deeper architecture that extracts redundant features due to inappropriate gradient flow. Among two RNN predictors, DRNN stands out with considerable performance margin in contrast to TextRNN predictor. In comparison with standalone CNN or RNN predictors, Hybrid predictor TextRCNN produces better performance because it reaps the benefit of both CNN and RNN architectures that extract comprehensive discriminative and contextual features. Two predictors namely region embedding and FastText performance remain in between the performance of top-performing hybrid predictor and least performing predictor AttentionConvNet. Overall, among all nine predictors, Fasttext-TextRCNN produces better performance across all three datasets. Moreover, following comprehensive performance analysis of nine distinct predictors and three word embeddings methods, the conclusion drawn in response to research question 5 indicates that generic word embeddings do not possess the capability to significantly improve the performance of deep learning predictors.

### 5.4 MLR-predictor and baseline predictors performance comparison

As discussed in Section 5.2, a large-scale experimental analysis of 108 data transformation-based predictive pipelines reveals that OkapiBM25-LP-LR predictive pipeline manages to produce highest performance over all three benchmark datasets. Similarly, among 12 algorithm adaption-based predictive pipelines, OkapiBM25-MLKNN achieves best performance across all three datasets. On the other hand as discussed in Section 5.2, from adapted deep learning predictors FastText pre-trained embeddings with hybrid predictor (Fasttext-TextRCNN) produces better performance across all three datasets. This section illustrates performance comparison of top-performing data transformation, algorithm adaption, and deep learning-based predictive pipelines using five different evaluation measures including: accuracy, precision, recall, f1-score and subset-accuracy.

**Figure 7:**
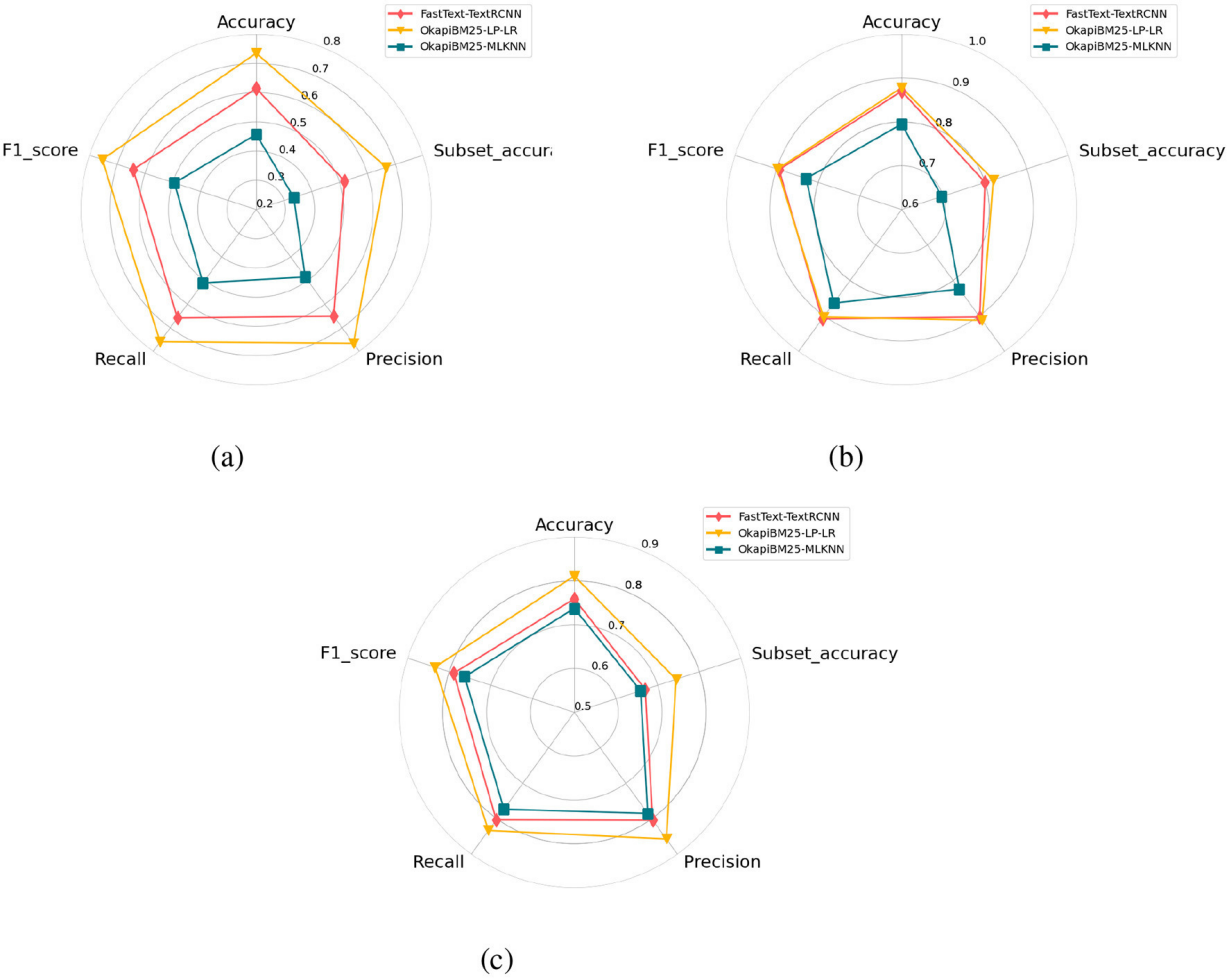
Performance comparison of adapted deep learning predictors based on different embeddings using three public benchmark dataset. **(a)** Promise. **(b)** EHR-Binary. **(c)** EHR-Multiclass.

It is evident from Figure 7 that among three top-performing predictors, FastText-TextRCNN predictor surpasses performance of OkapiBM25-MLKNN predictor by 15%, 6%, and 3% in terms of all five evaluation measures across promise, EHR-B, and EHR-M datasets, respectively. Proposed OkapiBM25-LP-LR predictor outperforms FastText-TextRCNN predictor with a significant margin of 19%, 1.5%, and 5% across promise, EHR-B, and EHR-M in terms of all evaluation matrices. Proposed OkapiBM25-LP-LR predictor outperforms OkapiBM25-MLKNN predictor with performance gain of ~26, 7, and 7% over promise, EHR-B, and EHR-M, respectively. Proposed OkapiBM25-LP-LR predictor utilizes comprehensive statistical representation and LP-based data transformation method capable of capturing co-relation among different classes. Thus, proposed predictor exhibits dominating performance over both algorithm adaption-based and deep learning-based top-performing predictors.

**Figure 8:**
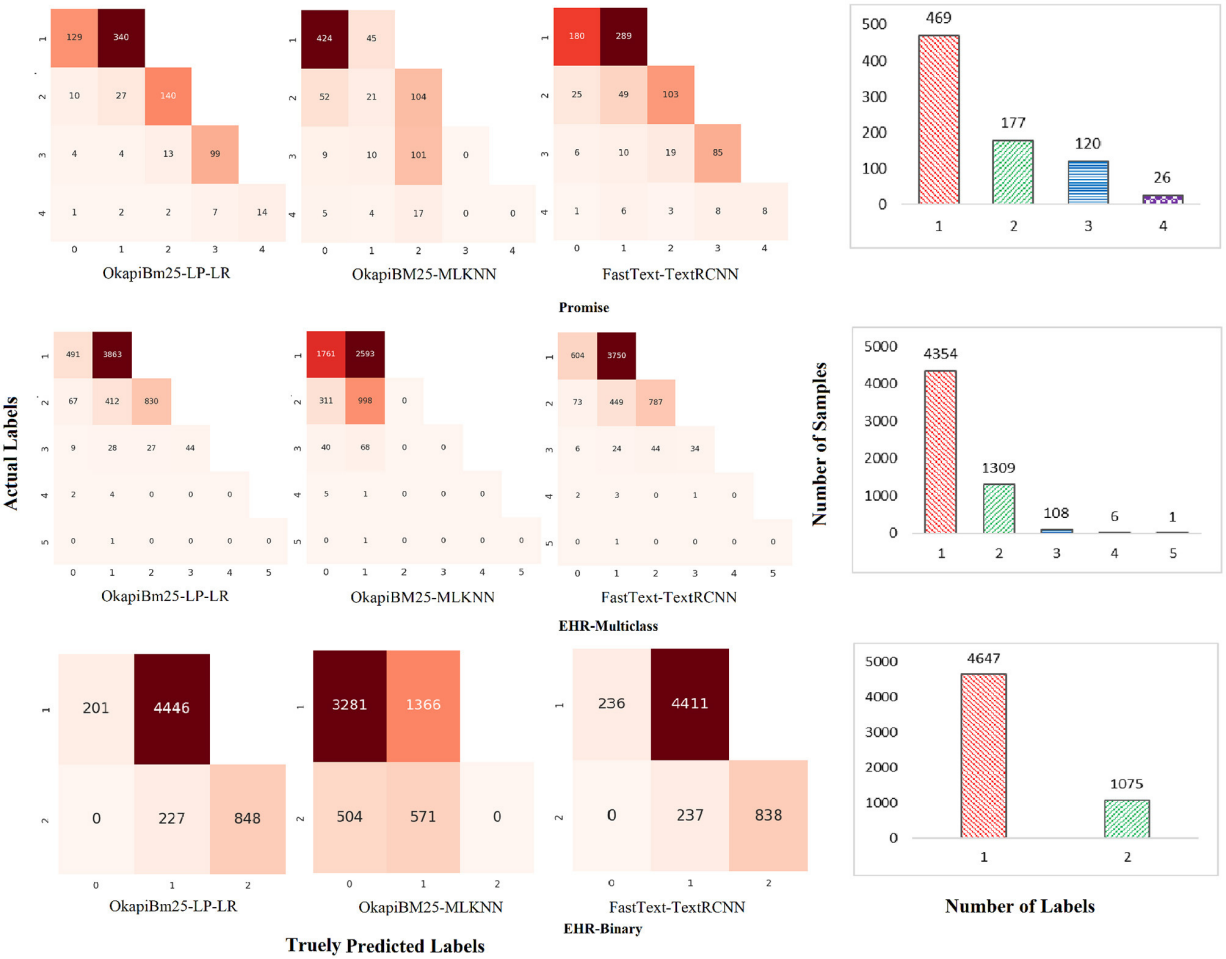
Performance analysis of top-performing predictors.

Furthermore, we assess and compare capabilities of three top-performing predictors for simultaneously accurately predicting different combinations of labels. Figure 8 graphically illustrates confusion matrices of actual and predicted combinations of labels and samples to labels combinations distribution across three benchmark datasets. It can be seen in Figure 8 top row confusion metrics and samples to labels distribution bar graph, over promise dataset, out of 469 uni-label samples 340 samples are correctly identified by proposed predictor. Contrarily, only 45 and 289 uni-label samples are correctly identified by OkapiBM25-MLKNN and Fasttext-TextRCNN predictors, respectively. Out of 170 bi-label samples, proposed predictor correctly identified 140 bi-labels while both baseline predictors correctly identified almost a similar number of bi-label samples, that is, 104. However unlike OkapiBM25-MLKNN predictor, FastText-TextRCNN predictor demonstrates twice effectiveness in correctly identifying at least one correct label among bi-label samples. Moreover, among 120 tri-labels samples proposed and OkapiBM25-MLKNN predictors correctly predicted 99 and 101 correct tri-label-related samples, while FastText-TextRCNN managed to predict only 85 correct samples. Among 26 tetra-label samples, proposed and FastText-TextRCNN predictors correctly identified 14 and 8 samples, respectively. On the other hand, OkapiBM25-MLKNN remains fail to correctly label any tetra-label samples. In a nutshell, proposed predictor remarkably outperformed other baseline predictors over uni-, bi-, tri-, and tetra-label samples. Furthermore, a similar performance trend also exists in other two datasets, namely, EHR-B and EHR-M.

**Figure 9:**
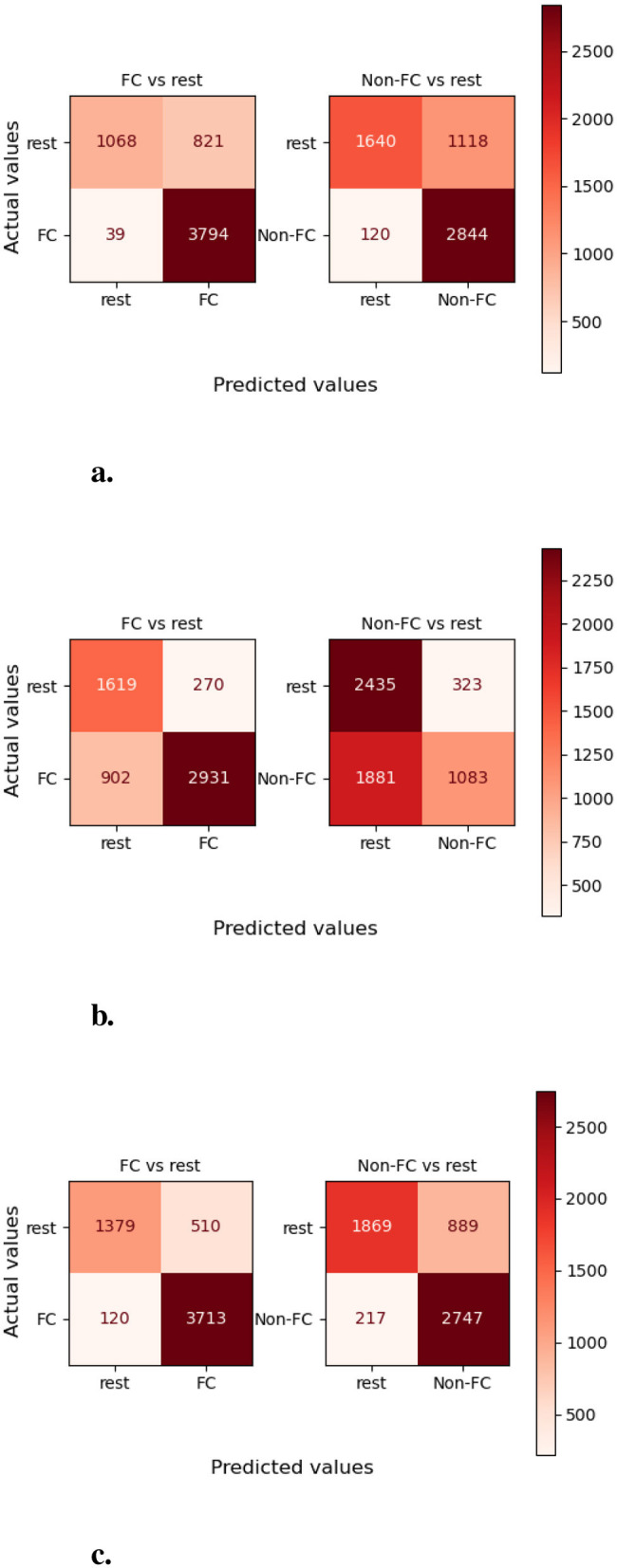
Class-wise performance analysis of MLR and baseline predictors in terms of number of correct and wrong predictions over EHR-Binary dataset. **(a)** OkapiBM25-LP-LR. **(b)** OkapiBM25-MLKNN. **(c)** FastText-TextRCNN.

Moreover, to assess and compare class-wise performance of proposed and baseline predictors, Figure 9 represents three predictors confusion matrices for EHR-Binary dataset. More specifically, this analysis aims to assess performance of proposed predictor when few classes contain a small number of samples as compared to other classes. For developing confusion matrices, we used one vs. all technique across all distinct classes, in which false positives, false negatives, true positives, and true negatives are calculated by considering one class as positive and all other classes as negative. Class-level performance analysis of proposed predictor reveals that ~98 and 96% samples of “functional” and “non-functional” classes are predicted correctly. Contrarily, the OkapiBM25-MLKNN predictor manages to correctly identifies ~76% samples of “functional” class and only 36% samples of “non-functional” class. FastText-TextRCNN predictor accurately identifies 73% and nearly 67% samples of “functional” and “non-functional” classes, respectively. Hence, it is evident that the proposed OkapiBM25-LP-LR predictor achieves the highest true positive rate for each individual class over promise dataset. In the Supplementary material, a detailed examination of Supplementary Figures 1, 2 demonstrates that all three predictors exhibit similar trends to those observed in the EHR-B dataset for the other two datasets promise and and EHR-M.

### 5.5 Performance comparison of proposed and state-of-the-art predictors

This section provides performance comparison of proposed and state-of-the-art multi-label requirements classification predictors across three benchmark datasets. It is evident from Table 7 state-of-the-art predictors are evaluated across three different evaluation measures namely macro precision, macro recall, and macro F1 measure. Moreover, Slankas and Williams (2013) reported all three measures performance for EHR-M dataset but only macro F1-score for promise dataset. To ensure fair performance comparison, we have also reported macro precision, macro recall, and macro F1-measure in this particular section. However, the remaining sections of the article focus on performance analysis primarily using the F1-score.

**Table 7:** Performance comparison of proposed and exiting multi-label requirements classification predictors over three benchmark datasets.

**Datasets**	**Precision**			**Recall**			**F1-score**		
	**Slankas and Williams (** **2013** **)**	**AlDhafer et al. (** **2022** **)**	**Proposed**	**Slankas and Williams (** **2013** **)**	**AlDhafer et al. (** **2022** **)**	**Proposed**	**Slankas and Williams (** **2013** **)**	**AlDhafer et al. (** **2022** **)**	**Proposed**
Promise	-	0.49	**0.552**	-	0.42	**0.590**	0.382	0.44	**0.570**
EHR- Binary	-	0.88	**0.889**	-	0.89	**0.909**	-	0.89	**0.899**
EHR- Multiclass	0.72	0.68	**0.558**	0.54	0.56	**0.766**	0.62	0.6	**0.645**

Bold values indicate performance values of proposed predictor.

Over promise dataset, among existing predictors, AlDhafer et al. (2022) predictor produces 6% better macro F1-score. However, over EHR-M dataset, Slankas and Williams (2013) predictor outperformed AlDhafer et al. (2022) predictor by 2%. On the other hand, over promise dataset, proposed predictor outperformed Slankas and Williams (2013) and AlDhafer et al. (2022) predictors with a significant performance margin of 19% and 13% in terms of macro F1-score. Similarly, over EHR-M dataset, proposed predictor outperformed Slankas and Williams (2013) and AlDhafer et al. (2022) predictors by performance margin of 2.5% and 4.5% in terms of F1-score. Over EHR-B dataset, proposed predictor outperform AlDhafer et al. (2022) predictor by a performance figure of 1%.

With respect to robustness, proposed and state-of-the-art predictors fall into two categories: highly biased and less-biased based on differences in precision and recall values. Highly-biased predictors have higher differences, and less-biased predictors have less difference between precision and recall values. It is evident from Table 7, Slankas and Williams (2013) predictor has almost 18% different between precision and recall. Although AlDhafer et al. (2022) predictor precision and recall performance figures are almost similar over EHR-B dataset. It has almost 7% precision and recall difference over promise dataset and ~14% performance difference in terms of precision and recall over EHR-M dataset. Based on precision and recall differences (greater than 5), state-of-the-art predictors (Slankas and Williams, 2013; AlDhafer et al., 2022) are baised toward type II error, over promise, and EHR-M dataset. Proposed predictor produced almost similar precision and recall values across promise and EHR-B; however, over EHR-M it is baised toward type I error.

AlDhafer et al. (2022) predictor less predictive performance is due to its reliability over deep learning architecture (BiGRU) that usually requires large training data to produce better performance and requirements classification datasets are smaller in size. Slankas and Williams (2013) predictor also could not manage to produce better performance although it makes use of traditional TFIDF representation learning approach along with binary relevance data transformation approach and SVM classifier. Binary relevance does not consider co-relations between labels and usually lacks in performance. Proposed predictor outperformed both state-of-the-art predictors with a significant performance margin because it makes use of more comprehensive representation learning method OkapiBM25 and data transformation method label powerset that considers correlations between class labels while transforming data from multi-label to multiclass. Another reason for producing better performance is proposed predictor reliability over swarm optimizer that smartly finds optimal values of hyper-parameters. It can be seen, Slankas and Williams (2013) predictive pipeline TFIDF-BR-SVM managed to produce 0.382% and 0.62% macro F1-score over promise and EHR-M datasets, respectively. However, in our experimentation, same predictive pipeline manged to produce 0.397% and 0.629% F1-score over promise and EHR-M datasets, respectively. A prime reason behind this performance boost is utilization of optimal hyper-parameters.

### 5.6 Case study

In addition to requirements classification, we also evaluated the effectiveness of the proposed MLR framework's top-performing predictive pipeline in handling the task of classifying customer reviews for various software products. With an aim to improve existing softwares by collecting users feedbacks from social media platforms, Jha and Mahmoud (2019) manually categorized users feedbacks into requirements four classes. In this study, the objective was to analyze how users are providing feedback about each requirement class. Primarily, 6,000 user reviews encompassing 2,369 instances of non-functional requirements and 3631 instances categorized as miscellaneous requirements. These reviews belong to various domains including games, communication, books, health, and more. The reviews are categorized into four distinct NFR classes namely dependability, performance, supportability, and usability.

More recently, Kaur and Kaur (2023) utilized aforementioned dataset for multi-label classification of reviews under two distinct experimental settings including (1) 10-fold cross-validation and (2) independent test set (70-30 split). The authors made use of binary relevance for data transformation along with BERT language model. The authors reported average F1-measure of 74 and 73 under first and second experimental setting, respectively. However, proposed predictor outperformed Kaur and Kaur (2023) predictor with a margin of 1.4 and 1% under first and second setting, respectively. The superior performance of proposed predictor reveals that it can be utilize to perform other different types of multi-label classification tasks related to software.

## 6 Limitations of study

This study covers a broad scope by investigating the potential of 124 machine learning and nine deep learning-based predictive pipelines. However, there are opportunities for further enhancements within these predictive pipelines. For instance, the study utilizes pre-trained word embeddings generated from generic textual data. Performance of predictive pipelines might improve significantly if the word embeddings were generated specifically from requirements data. Additionally, the study examines only two types of word embeddings, namely, word2vec and FastText. Other methods, such as deepwalk, graph representation, node2vec, LINE, and HOPE, remain unexplored. These alternative methods could potentially enhance the performance of the predictive pipelines. Furthermore, while this study includes a case study comparing the performance of the proposed predictive pipeline with the BERT language model, it does not explore the potential of large language models for requirements classification task.

## 7 Conclusion

To empower multi-label requirements classification process, the article in hand presents a versatile computational framework named MLR-Predictor. With an aim to transform requirements into statistical vectors having discriminative patterns among different classes, MLR-Predictor is enriched with diverse types of words embeddings and a unique encoder Okapi BM25. Furthermore, it is strengthened with four algorithm adaptation and three data transformation methods along with nine machine learning classifiers. To find optimal hyper-parameters of classifiers and Okapi BM25 encoder, MLR-Predictor is empowered with swarm optimizer. MLR-Predictor distinct predictive pipelines and nine adapted deep learning predictors are evaluated on three benchmark datasets using eight different evaluation measures. The performance analysis of diverse predictive pipelines within the proposed framework reveals that, at the representation level, the Okapi BM25 method is the most effective among four different representation learning approaches namely TFIDF, word2vec, fasttext, and glove.It transforms requirements into a statistical feature space by assigning scores to words based on their actual discriminative potential. Furthermore, among three data transformation approaches, the Label Powerset method is particularly effective in converting multi-label data into multiclass data. Among four algorithm adaption-based methods, xx method produced better performance. Overall, among 124 machine learning-based predictive pipelines, OKAPI-BM25 representation learning, Label powerset data transformation, and LR classifier-based predictive pipeline produced best performance. On the other hand among adapted nine different deep learning predictors, okapiBM25-LP-LR predictor produced highest performance. Furthermore, deep learning predictors remain fail to surpass the performance of traditional machine learning-based predictive pipelines, primarily due to the limited size of the training data. Furthermore, in comparison with state-of-the-art requirements classification predictor, the OkapiBM25-LPLR predictive pipeline demonstrated enhancements in macro F1-scores of 13%, 1.5%, and 2.5% across the promise, EHR-B, and EHR-M datasets, respectively.

Promising future directions for this study include accumulation of a substantial amount of requirements-related data along with time and space complexity of MLR framework. Additionally, investigating the potential of large language models (LLMs) and exploring predictor's robustness against adversarial attacks (Kwon and Lee, 2023, 2024) would be crucial to ensure its reliability in real-world applications. These advancements would not only strengthen the current approach but also pave the way for more sophisticated and resilient requirements classification systems, potentially revolutionizing software engineering practices.

## Data Availability

The dataset is publically available at https://github.com/SummraSaleem94/MLR-predictor-framework-for-multi-label-requirements-classification/.

## Footnotes

^1^ http://vectors.nlpl.eu/repository/

^2^ https://scikit-learn.org/

^3^ https://numpy.org/

^4^ https://docs.python.org/3/library/math.html

^5^ https://scipy.org/

^6^ https://pandas.pydata.org/

^7^ https://matplotlib.org/

^8^ https://www.fast.ai/

^9^ https://pytorch.org/
