# Supplementary material for: MLR-predictor: a versatile and efficient computational framework for multi-label requirements classification
Source: Front Artif Intell. 2024 Nov 27;7:1481581. doi: 10.3389/frai.2024.1481581 (PMC11632133; doi:10.3389/frai.2024.1481581)
Supplement: Supplementary file 1 [file Data_Sheet_1.pdf]

Supplementary Material

1 ADAPTED DEEP LEARNING PREDICTORS

1.1 TextCNN

We adapted Yoon Kim et al. predictor (1) that has achieved promising performance for sentence classification, sentimental analysis and question classification tasks. Following working paradigm of CNN architectures in computer vision domain (2), authors proposed a multichannel strategy based CNN predictor where at each channel they provide same input samples but each channel contains different embedding type and filter kernel size that helps in extracting different types of features. In proposed multichannel predictor, apart from extracting different types of features, authors took advantage of multiple channels to comprehensively train predictor by fine-tuning embeddings at one channel known as dynamic channel and keeping other embedding channel static. In this work, we utilized pretrained embeddings (3) at static channel and randomly initialized embeddings at dynamic channel. Feature representations from both static and dynamic channels are passed to 3 convolutional layers having 100 filters of size 3, 4 and 5. Aggregated output of these convolution layers is passed to another convolutional layer that extracts more discriminative features. Afterward, max-pooling is applied on extracted features to remove irrelevant and redundant features. Further, features are flattened before passing to dense layer followed by dropout layer (4) with probability of 0.5. Finally, a dense layer performs classification.

1.2 DPCNN

Armand Joulin et al. (5) proposed Deep Pyramid CNN (DPCNN), a deep word-level CNN predictor. The authors designed a deeper CNN architecture to boost classifier performance while considering computational cost. For tasks including topic classification (6) and sentence classification (7) the proposed predictor exhibits superior performance over existing methods. We designed a deep predictor for multi-label classification based on DPCNN architecture. Our adapted model comprises three CNN blocks, each of which contains two consecutive CNN layers where each layer has 16 kernels with filter size of 3. The first CNN block is introduced after the embedding layer. Input and output of first CNN block are aggregated and fed to max pooling layer followed by second CNN block. Combined features of max-pooling layer and second CNN block are passed to third CNN block. Extracted features of max pooling and third CNN block are aggregated and fed to final max-pooling layer. Finally, linear layer serving as classifier receives output of preceding max-pooling layer.

1.3 VDCNN

Alexis et al. (8) drew inspiration from promising performance of deep CNN architectures in computer vision (9) (10) and suggested Very Deep Convolutional Neural Network (VDCNN) comprising 29 convolutional layers to capture comprehensive hierarchical representations of phrases. The proposed predictor utilizes tiny convolution filters and character-level knowledge of text. Authors claimed that classification performance boosted significantly after increasing network depth. We also designed a deep CNN predictor for multi-label classification, which utilizes convolution layer with 64 kernels each with filter size of 3 to extract discriminative features. This convolution layer is followed by set of 4 convolution blocks, while each block has 2 convolution layers with 64 kernels and a batch normalization layer that rescales extracted features. Afterward, output of last convolution block is fed to max-pooling layer. Two

fully connected layers with a total of 2048 neurons in each layer receive the output of max-pooling layer. The final linear layer acting as classifier receives output of preceding fully connected layer.

1.4 AttentiveConvNet

Wenpeng Yin et al. (11) proposed attentiveConvNet predictor that utilizes convolution operation and attention module to extract significant features for each word based on local and global contextual information. According to authors, attentiveConvNet demonstrated encouraging results for various NLP tasks (12; 13) by learning comprehensive sentence representation along with contextual information. We adapted this predictor for multi-label requirement classification. Our predictor comprises an embedding layer, followed by 5 highway layers namely; xcontext highway, x-self highway, a-context highway, a-self highway and beneficiary highway. Each highway includes two convolution layers in series along with sigmoid (14) and tanh (15) activation functions. The bilinear attention layer receives output of final highway layer. The output of this attention layer is fed to two dense layers, followed by pooling layer. Finally, output features of pooling layer and two dense layers are merged and fed to linear layer.

1.5 TextRNN

Leveraging the success of TextRNN method (16) for multi-class text classification we designed deep learning-based predictive pipeline identical to TextRNN. We designed three modifications of adapted predictor by using different versions of RNN family. TextRNN predictor makes use of RNN cells, while TextLSTM and TextGRU predictors utilize LSTM and GRU layers in their architectures, respectively. All three predictors begin with embedding layer followed by specific layer of each RNN family (RNN, LSTM and GRU) and attention module. Lastly, attention module's output is forwarded to linear layer that serves as classifier.

1.6 DRNN

Wang et al. (17) presented a Disconnected Recurrent Neural Network (DRNN) predictor to incorporate position invariance in conventional RNN. Every time step's hidden state is limited to reflect adjacent words of current position by controlling the length of information flow. We adapted this predictor to benchmark performance of multi-label classification. In this predictor (17), output of embedding layer flows to dropout layer with dropout rate of 0.1 followed by two bidirectional RNN layers. Similar to TextRNN predictor, we utilized RNN family, to develop three distinct variations of same predictor. These predictors make use of RNN, LSTM and GRU and named as DRNN, DLSTM and DGRU, respectively. We choose the ideal number of hidden neurons in adapted predictors: ranging from 5 to 100 units. Two linear layers are added after the output of the second RNN is transferred to the batch normalization layer. The first linear layer comprises 10 neurons and output is passed to final linear layer that serves as classifier.

1.7 TextRCNN

It is believed that RNNs are more suitable to capture long-range contextual information, although CNNs are proficient at extracting location invariant and local features. Siwei lai et al. (18) presented hybrid predictor called Recurrent Convolutional Neural Network (RCNN) based on bi-directional recurrent structure to extract contextual information and leveraged neural word representation and max pooling layer to acquire the most discriminative words. Authors claimed that proposed hybrid predictor outperformed standalone CNN and recursive RNN predictors. We developed three distinct predictors for classifying multi-label requirements in accordance with architecture proposed by Siwei lai et al. (18). Our adapted predictor comprises a recurrent layer followed by three consecutive CNN layers with kernel sizes of 2,

3 and 4 for the first, second, and third layers, respectively. A max pooling layer is applied after CNN layer followed by a linear layer. In one of our predictor's variations, we incorporated RNN cells and named predictor as TextRNN-CNN. In another variation, we utilized LSTM cells and named predictor as TextLSTM-CNN. Finally, in our third variation, we employed GRU cells and named the predictor TextGRU-CNN.

1.8 Region Embedding

Qiao et al. (19) suggested region embedding technique to develop task-specific representations of n-grams. Researchers found that region embeddings efficiently captured phrasal expression of text and performed better than simple word embeddings for text classification on several benchmark datasets. After the success of region embedding approach, we adapted it for multi-label text classification. The region embedding predictor begins with a region embedding layer having 64 dimensions and dropout rate of 0.3. The output of region embedding layer is fed to final linear layer.

1.9 FastText

Joulin et al. (20) proposed a deep learning predictor that utilizes random embeddings and updates them during training based on back-propagation loss. According to authors (20), the proposed predictor not only outperformed other deep learning-based predictors but also reported fast processing speed. We modified this predictor to perform multi-label requirement classification. The predictor begins with layer of random embeddings followed by linear layer that serves as classifier.

Table S1. Performance analysis of data transformation and algorithm adaptation based requirements multilabel classification predictive pipelines using TFIDF representation method and classifiers optimal hyper-parameters over EHR-Binary dataset

Problem Transformation	Classifier	Accuracy	Subset Accuracy	Precision	Micro Precision	Macro Precision	Recall	Micro Recall	Macro Recall	F1 Score	Micro F1	Macro F1	Average Precision	Hamming Loss	Ranking Loss
Binary Relevance	AB	0.776	0.646	0.811	0.859	0.854	0.870	0.796	0.790	0.819	0.826	0.821	0.617	0.215	0.280
	DT	0.773	0.670	0.806	0.838	0.832	0.845	0.833	0.828	0.808	0.835	0.830	0.578	0.196	0.260
	ET	0.848	0.776	0.879	0.880	0.873	0.890	0.890	0.887	0.873	0.885	0.880	0.573	0.136	0.160
	GB	0.803	0.655	0.827	0.918	0.913	0.927	0.788	0.783	0.852	0.848	0.843	0.639	0.196	0.297
	LR	0.869	0.784	0.897	0.914	0.908	0.926	0.873	0.868	0.897	0.893	0.888	0.603	0.130	0.160
	NB	0.855	0.746	0.883	0.923	0.914	0.936	0.848	0.847	0.892	0.883	0.879	0.596	0.145	0.198
	RF	0.825	0.741	0.857	0.866	0.858	0.877	0.870	0.865	0.853	0.868	0.862	0.572	0.157	0.191
	SVC	0.594	0.188	0.594	1.000	1.000	1.000	0.594	0.594	0.729	0.745	0.745	0.835	0.406	0.812
	XGB	0.848	0.760	0.874	0.902	0.897	0.910	0.859	0.855	0.877	0.880	0.876	0.603	0.146	0.188
Label Powerset	AB	0.754	0.679	0.806	0.768	0.753	0.776	0.809	0.803	0.779	0.788	0.777	0.547	0.246	0.216
	DT	0.810	0.739	0.849	0.833	0.827	0.841	0.845	0.840	0.833	0.839	0.833	0.588	0.190	0.182
	ET	0.858	0.806	0.900	0.853	0.843	0.868	0.903	0.902	0.875	0.877	0.871	0.555	0.142	0.110
	GB	0.824	0.767	0.874	0.818	0.807	0.833	0.879	0.876	0.844	0.847	0.840	0.551	0.176	0.134
	LR	0.868	0.814	0.913	0.860	0.853	0.879	0.913	0.909	0.887	0.886	0.880	0.568	0.132	0.097
	NB	0.846	0.781	0.907	0.825	0.806	0.852	0.908	0.912	0.868	0.864	0.856	0.521	0.154	0.099
	RF	0.832	0.780	0.876	0.829	0.818	0.841	0.881	0.880	0.850	0.854	0.848	0.551	0.168	0.133
	SVC	0.576	0.482	0.670	0.564	0.500	0.576	0.670	0.335	0.607	0.612	0.401	0.335	0.424	0.330
	XGB	0.861	0.811	0.899	0.862	0.856	0.874	0.900	0.897	0.878	0.881	0.876	0.575	0.139	0.114
Classifier Chain	AB	0.788	0.719	0.835	0.802	0.789	0.811	0.836	0.834	0.812	0.819	0.811	0.551	0.212	0.187
	DT	0.805	0.733	0.844	0.831	0.825	0.839	0.839	0.835	0.829	0.835	0.830	0.586	0.195	0.189
	ET	0.862	0.807	0.900	0.865	0.855	0.878	0.904	0.902	0.880	0.884	0.878	0.561	0.136	0.115
	GB	0.785	0.724	0.837	0.781	0.756	0.793	0.845	0.861	0.805	0.812	0.805	0.495	0.215	0.171
	LR	0.863	0.809	0.908	0.854	0.839	0.872	0.909	0.911	0.881	0.881	0.874	0.539	0.137	0.101
	NB	0.843	0.756	0.890	0.863	0.846	0.883	0.872	0.881	0.872	0.867	0.863	0.537	0.157	0.151
	RF	0.842	0.778	0.884	0.847	0.836	0.862	0.886	0.885	0.863	0.866	0.860	0.555	0.156	0.135
	SVC	0.594	0.188	0.594	1.000	1.000	1.000	0.594	0.594	0.729	0.745	0.745	0.835	0.406	0.812
	XGB	0.850	0.796	0.887	0.858	0.846	0.868	0.886	0.885	0.868	0.872	0.865	0.558	0.150	0.131
Algorithm Adaptation Approaches	BRKNNa	0.792	0.729	0.825	0.808	0.799	0.820	0.827	0.824	0.813	0.817	0.761	0.428	0.203	0.202
	BRKNNb	0.801	0.768	0.831	0.811	0.804	0.822	0.831	0.827	0.819	0.821	0.815	0.508	0.199	0.185
	MLKNN	0.810	0.717	0.816	0.824	0.830	0.914	0.802	0.797	0.814	0.813	0.808	0.500	0.209	0.226
	MLARAM	0.576	0.482	0.670	0.564	0.500	0.576	0.670	0.335	0.607	0.612	0.401	0.335	0.424	0.330

2 CLASS-WISE PERFORMANCE ANALYSIS OF MLR AND BASELINE PREDICTORS

Table S2. Performance analysis of data transformation and algorithm adaptation based requirements multilabel classification predictive pipelines using TFIDF representation method and classifiers optimal hyper-parameters over EHR-Multiclass dataset

Problem Transformation	Classifier	Accuracy	Subset Accuracy	Precision	Micro Precision	Macro Precision	Recall	Micro Recall	Macro Recall	F1 Score	Micro F1	Macro F1	Average Precision	Hamming Loss	Ranking Loss
Binary Relevance	AB	0.677	0.578	0.730	0.700	0.447	0.723	0.805	0.680	0.710	0.749	0.538	0.070	0.040	0.284
	DT	0.687	0.569	0.728	0.754	0.550	0.766	0.760	0.583	0.727	0.756	0.564	0.077	0.041	0.247
	ET	0.707	0.643	0.760	0.695	0.359	0.719	0.904	0.790	0.728	0.785	0.492	0.063	0.032	0.283
	GB	0.710	0.610	0.770	0.723	0.474	0.750	0.805	0.666	0.744	0.762	0.553	0.077	0.038	0.256
	LR	0.658	0.589	0.721	0.641	0.192	0.666	0.902	0.521	0.681	0.749	0.280	0.059	0.036	0.335
	NB	0.616	0.542	0.692	0.586	0.115	0.618	0.859	0.264	0.641	0.696	0.160	0.057	0.043	0.382
	RF	0.676	0.611	0.732	0.664	0.286	0.687	0.889	0.700	0.698	0.760	0.405	0.062	0.035	0.314
	SVC	0.574	0.479	0.674	0.533	0.067	0.574	0.673	0.045	0.606	0.595	0.054	0.070	0.061	0.426
	XGB	0.725	0.653	0.773	0.730	0.397	0.748	0.872	0.712	0.749	0.794	0.508	0.069	0.032	0.255
Label Powerset	AB	0.578	0.483	0.678	0.537	0.074	0.578	0.678	0.088	0.611	0.599	0.076	0.070	0.061	0.422
	DT	0.698	0.623	0.742	0.716	0.425	0.729	0.731	0.480	0.723	0.724	0.450	0.070	0.046	0.276
	ET	0.756	0.695	0.809	0.744	0.410	0.766	0.817	0.758	0.777	0.779	0.531	0.075	0.036	0.235
	GB	0.710	0.636	0.762	0.712	0.436	0.734	0.750	0.545	0.736	0.730	0.483	0.073	0.044	0.270
	LR	0.725	0.658	0.785	0.705	0.272	0.733	0.795	0.591	0.747	0.747	0.372	0.076	0.040	0.268
	NB	0.626	0.551	0.704	0.595	0.115	0.628	0.715	0.244	0.652	0.649	0.156	0.073	0.054	0.372
	RF	0.729	0.668	0.784	0.714	0.343	0.738	0.794	0.672	0.750	0.752	0.453	0.075	0.040	0.264
	SVC	0.574	0.479	0.674	0.533	0.067	0.574	0.673	0.045	0.606	0.595	0.054	0.070	0.061	0.426
	XGB	0.739	0.672	0.788	0.739	0.398	0.760	0.788	0.633	0.762	0.762	0.486	0.074	0.039	0.243
Classifier Chain	AB	0.687	0.598	0.742	0.700	0.452	0.722	0.791	0.671	0.718	0.743	0.539	0.067	0.041	0.283
	DT	0.691	0.587	0.732	0.742	0.549	0.755	0.744	0.574	0.727	0.743	0.560	0.073	0.043	0.255
	ET	0.722	0.661	0.773	0.711	0.373	0.732	0.905	0.789	0.742	0.796	0.505	0.065	0.031	0.270
	GB	0.713	0.624	0.773	0.716	0.466	0.742	0.810	0.679	0.743	0.760	0.552	0.069	0.038	0.263
	LR	0.717	0.644	0.785	0.696	0.273	0.724	0.876	0.600	0.742	0.775	0.374	0.071	0.034	0.277
	NB	0.622	0.543	0.703	0.589	0.120	0.624	0.855	0.334	0.649	0.697	0.176	0.057	0.043	0.376
	RF	0.686	0.623	0.742	0.669	0.295	0.693	0.889	0.703	0.707	0.763	0.415	0.064	0.035	0.308
	SVC	0.574	0.479	0.674	0.533	0.067	0.574	0.673	0.045	0.606	0.595	0.054	0.070	0.061	0.426
	XGB	0.747	0.683	0.797	0.743	0.408	0.762	0.856	0.696	0.768	0.795	0.513	0.069	0.032	0.240
Algorithm Adaptation Approaches	BRKNNa	0.720	0.656	0.784	0.711	0.357	0.731	0.897	0.705	0.741	0.793	0.473	0.061	0.031	0.270
	BRKNNb	0.009	0.006	0.005	0.012	0.077	0.010	0.012	0.055	0.011	0.012	0.059	0.003	0.164	0.990
	MLKNN	0.741	0.687	0.781	0.746	0.488	0.763	0.835	0.709	0.763	0.783	0.584	0.066	0.032	0.232
	MLARAM	0.574	0.479	0.688	0.533	0.067	0.574	0.673	0.045	0.606	0.595	0.054	0.070	0.061	0.426

Table S3. Performance analysis of data transformation and algorithm adaptation based requirements multilabel classification predictive pipelines using TFIDF representation method and classifiers optimal hyper-parameters over Promise dataset

Problem Transformation	Classifier	Accuracy	Subset Accuracy	Precision	Micro Precision	Macro Precision	Recall	Micro Recall	Macro Recall	F1 Score	Micro F1	Macro F1	Average Precision	Hamming Loss	Ranking Loss
Binary Relevance	AB	0.463	0.324	0.530	0.565	0.391	0.529	0.717	0.514	0.507	0.631	0.443	0.056	0.067	0.483
	DT	0.492	0.325	0.558	0.622	0.458	0.594	0.652	0.467	0.547	0.636	0.460	0.065	0.071	0.424
	ET	0.460	0.377	0.519	0.537	0.311	0.476	0.891	0.530	0.484	0.669	0.391	0.049	0.054	0.526
	GB	0.452	0.344	0.524	0.525	0.371	0.486	0.795	0.536	0.487	0.631	0.437	0.051	0.062	0.521
	LR	0.273	0.189	0.348	0.371	0.121	0.278	0.924	0.305	0.298	0.528	0.172	0.034	0.067	0.722
	NB	0.251	0.161	0.329	0.358	0.098	0.254	0.925	0.172	0.277	0.515	0.125	0.033	0.068	0.746
	RF	0.366	0.276	0.444	0.445	0.215	0.376	0.885	0.444	0.394	0.589	0.288	0.041	0.062	0.626
	SVC	0.457	0.335	0.485	0.546	0.351	0.510	0.692	0.461	0.494	0.608	0.397	0.057	0.071	0.499
	XGB	0.413	0.317	0.476	0.511	0.270	0.438	0.773	0.423	0.442	0.613	0.328	0.049	0.065	0.567
Label Powerset	AB	0.345	0.185	0.418	0.496	0.155	0.386	0.409	0.117	0.388	0.448	0.133	0.062	0.124	0.620
	DT	0.452	0.386	0.491	0.522	0.325	0.479	0.518	0.312	0.474	0.520	0.317	0.054	0.097	0.526
	ET	0.616	0.551	0.652	0.669	0.455	0.636	0.692	0.457	0.635	0.680	0.454	0.063	0.064	0.368
	GB	0.579	0.501	0.615	0.677	0.445	0.610	0.625	0.464	0.603	0.649	0.453	0.067	0.074	0.396
	LR	0.619	0.542	0.643	0.716	0.351	0.656	0.648	0.375	0.640	0.680	0.362	0.070	0.068	0.351
	NB	0.557	0.460	0.578	0.704	0.295	0.608	0.559	0.269	0.583	0.623	0.280	0.074	0.087	0.401
	RF	0.541	0.474	0.575	0.622	0.350	0.564	0.628	0.357	0.560	0.624	0.353	0.063	0.076	0.440
	SVC	0.312	0.152	0.382	0.478	0.125	0.353	0.386	0.048	0.354	0.427	0.070	0.060	0.130	0.653
	XGB	0.529	0.454	0.567	0.621	0.359	0.555	0.609	0.381	0.551	0.614	0.368	0.060	0.079	0.450
Classifier Chain	AB	0.469	0.366	0.508	0.568	0.397	0.535	0.656	0.458	0.503	0.608	0.424	0.055	0.074	0.477
	DT	0.496	0.373	0.528	0.605	0.447	0.586	0.645	0.465	0.535	0.624	0.454	0.064	0.073	0.432
	ET	0.475	0.414	0.509	0.564	0.316	0.489	0.893	0.529	0.492	0.690	0.395	0.050	0.051	0.514
	GB	0.489	0.425	0.521	0.566	0.390	0.519	0.807	0.545	0.509	0.664	0.454	0.054	0.058	0.487
	LR	0.383	0.322	0.401	0.509	0.209	0.409	0.844	0.365	0.398	0.633	0.264	0.047	0.059	0.596
	NB	0.303	0.239	0.326	0.459	0.140	0.320	0.862	0.193	0.319	0.598	0.161	0.040	0.062	0.682
	RF	0.372	0.310	0.415	0.446	0.233	0.385	0.854	0.476	0.391	0.583	0.311	0.040	0.064	0.617
	SVC	0.468	0.384	0.512	0.540	0.367	0.507	0.668	0.444	0.495	0.596	0.400	0.053	0.074	0.501
	XGB	0.439	0.365	0.475	0.541	0.288	0.465	0.744	0.412	0.460	0.625	0.337	0.050	0.065	0.540
Algorithm Adaptation Approaches	BRKNNa	0.439	0.371	0.437	0.541	0.244	0.456	0.823	0.433	0.457	0.652	0.311	0.048	0.058	0.548
	BRKNNb	0.070	0.031	0.071	0.101	0.113	0.099	0.098	0.076	0.089	0.100	0.087	0.012	0.184	0.908
	MLKNN	0.431	0.345	0.413	0.519	0.258	0.466	0.668	0.357	0.456	0.584	0.300	0.049	0.674	0.540
	MLARAM	0.312	0.152	0.394	0.478	0.125	0.353	0.386	0.048	0.354	0.427	0.070	0.060	0.130	0.653

Table S4. Performance analysis of data transformation and algorithm adaptation based requirements multilabel classification predictive pipelines using OkapiBm25 representation method and classifiers optimal hyper-parameters over EHR-Binary dataset

Problem Transformation	Classifier	Accuracy	Subset Accuracy	Precision	Micro Precision	Macro Precision	Recall	Micro Recall	Macro Recall	F1 Score	Micro F1	Macro F1	Average Precision	Hamming Loss	Ranking Loss
Binary Relevance	AB	0.816	0.699	0.847	0.893	0.886	0.903	0.819	0.814	0.855	0.854	0.849	0.611	0.181	0.240
	DT	0.762	0.656	0.796	0.828	0.820	0.836	0.827	0.821	0.798	0.827	0.821	0.570	0.206	0.270
	ET	0.855	0.772	0.875	0.907	0.903	0.916	0.839	0.846	0.882	0.877	0.873	0.578	0.144	0.186
	GB	0.870	0.793	0.894	0.915	0.910	0.924	0.882	0.878	0.896	0.899	0.894	0.597	0.123	0.159
	LR	0.870	0.797	0.898	0.904	0.897	0.915	0.890	0.884	0.894	0.897	0.891	0.592	0.124	0.146
	RF	0.866	0.775	0.888	0.926	0.921	0.935	0.860	0.856	0.896	0.892	0.887	0.612	0.134	0.181
	SVC	0.859	0.750	0.877	0.942	0.940	0.949	0.844	0.840	0.895	0.890	0.887	0.643	0.138	0.214
	XGB	0.859	0.778	0.885	0.905	0.901	0.914	0.871	0.867	0.886	0.888	0.883	0.601	0.136	0.169
Label Powerset	AB	0.811	0.740	0.863	0.813	0.799	0.829	0.861	0.857	0.834	0.836	0.827	0.548	0.189	0.156
	DT	0.800	0.728	0.840	0.826	0.819	0.833	0.836	0.831	0.824	0.831	0.825	0.586	0.200	0.193
	ET	0.879	0.829	0.921	0.870	0.861	0.887	0.922	0.922	0.896	0.895	0.890	0.557	0.121	0.087
	GB	0.874	0.816	0.908	0.887	0.880	0.899	0.901	0.897	0.894	0.894	0.888	0.582	0.126	0.116
	LR	0.887	0.834	0.919	0.895	0.889	0.908	0.913	0.909	0.905	0.904	0.899	0.584	0.113	0.101
	RF	0.864	0.815	0.907	0.855	0.846	0.870	0.911	0.909	0.880	0.882	0.876	0.558	0.136	0.100
	SVC	0.874	0.819	0.907	0.882	0.876	0.895	0.891	0.896	0.892	0.891	0.886	0.572	0.127	0.115
	XGB	0.875	0.828	0.909	0.878	0.872	0.889	0.909	0.906	0.891	0.893	0.889	0.577	0.125	0.105
Classsifier Chain	AB	0.848	0.787	0.886	0.858	0.847	0.870	0.882	0.880	0.868	0.870	0.863	0.563	0.152	0.136
	DT	0.803	0.727	0.842	0.831	0.825	0.838	0.836	0.830	0.828	0.833	0.828	0.591	0.198	0.194
	ET	0.876	0.827	0.912	0.876	0.862	0.888	0.912	0.916	0.892	0.893	0.888	0.546	0.124	0.101
	GB	0.870	0.819	0.905	0.876	0.865	0.887	0.903	0.902	0.888	0.889	0.883	0.559	0.130	0.112
	LR	0.879	0.820	0.912	0.892	0.884	0.904	0.903	0.899	0.898	0.898	0.891	0.577	0.121	0.114
	RF	0.865	0.813	0.898	0.875	0.863	0.885	0.896	0.898	0.883	0.885	0.880	0.553	0.135	0.122
	SVC	0.877	0.820	0.910	0.888	0.880	0.900	0.903	0.899	0.896	0.895	0.889	0.575	0.123	0.114
	XGB	0.863	0.812	0.897	0.869	0.860	0.879	0.896	0.895	0.880	0.883	0.877	0.564	0.137	0.119
Algorithm Adaptation Appraoches	BRKNNa	0.766	0.655	0.797	0.836	0.816	0.841	0.784	0.794	0.802	0.809	0.805	0.546	0.235	0.274
	BRKNNb	0.801	0.730	0.814	0.832	0.821	0.832	0.831	0.831	0.824	0.836	0.822	0.557	0.199	0.199
	MLKNN	0.795	0.696	0.824	0.858	0.852	0.864	0.810	0.805	0.829	0.834	0.828	0.605	0.204	0.241
	MLARAM	0.714	0.620	0.867	0.680	0.660	0.714	0.808	0.853	0.746	0.739	0.744	0.508	0.286	0.192

Table S5. Performance analysis of data transformation and algorithm adaptation based requirements multilabel classification predictive pipelines using OkapiBm25 representation method and classifiers optimal hyper-parameters over EHR-Multiclass dataset

Problem Transformation	Classifier	Accuracy	Subset Accuracy	Precision	Micro Precision	Macro Precision	Recall	Micro Recall	Macro Recall	F1 Score	Micro F1	Macro F1	Average Precision	Hamming Loss	Ranking Loss
Binary Relevance	AB	0.677	0.578	0.730	0.700	0.447	0.723	0.805	0.680	0.710	0.749	0.538	0.070	0.040	0.284
	DT	0.687	0.569	0.728	0.754	0.550	0.766	0.760	0.583	0.727	0.756	0.564	0.077	0.041	0.247
	ET	0.707	0.643	0.760	0.695	0.359	0.719	0.904	0.790	0.728	0.785	0.492	0.063	0.032	0.283
	GB	0.710	0.610	0.770	0.723	0.474	0.750	0.805	0.666	0.744	0.762	0.553	0.077	0.038	0.256
	LR	0.658	0.589	0.721	0.641	0.192	0.666	0.902	0.521	0.681	0.749	0.280	0.059	0.036	0.335
	NB	0.616	0.542	0.692	0.586	0.115	0.618	0.859	0.264	0.641	0.696	0.160	0.057	0.043	0.382
	RF	0.676	0.611	0.732	0.664	0.286	0.687	0.889	0.700	0.698	0.760	0.405	0.062	0.035	0.314
	SVC	0.574	0.479	0.674	0.533	0.067	0.574	0.673	0.045	0.606	0.595	0.054	0.070	0.061	0.426
	XGB	0.725	0.653	0.773	0.730	0.397	0.748	0.872	0.712	0.749	0.794	0.508	0.069	0.032	0.255
Label Powerset	AB	0.578	0.483	0.678	0.537	0.074	0.578	0.678	0.088	0.611	0.599	0.076	0.070	0.061	0.422
	DT	0.698	0.623	0.742	0.716	0.425	0.729	0.731	0.480	0.723	0.724	0.450	0.070	0.046	0.276
	ET	0.756	0.695	0.809	0.744	0.410	0.766	0.817	0.758	0.777	0.779	0.531	0.075	0.036	0.235
	GB	0.710	0.636	0.762	0.712	0.436	0.734	0.750	0.545	0.736	0.730	0.483	0.073	0.044	0.270
	LR	0.725	0.658	0.785	0.705	0.272	0.733	0.795	0.591	0.747	0.747	0.372	0.076	0.040	0.268
	NB	0.626	0.551	0.704	0.595	0.115	0.628	0.715	0.244	0.652	0.649	0.156	0.073	0.054	0.372
	RF	0.729	0.668	0.784	0.714	0.343	0.738	0.794	0.672	0.750	0.752	0.453	0.075	0.040	0.264
	SVC	0.574	0.479	0.674	0.533	0.067	0.574	0.673	0.045	0.606	0.595	0.054	0.070	0.061	0.426
	XGB	0.739	0.672	0.788	0.739	0.398	0.760	0.788	0.633	0.762	0.762	0.486	0.074	0.039	0.243
Classifier Chain	AB	0.687	0.598	0.742	0.700	0.452	0.722	0.791	0.671	0.718	0.743	0.539	0.067	0.041	0.283
	DT	0.691	0.587	0.732	0.742	0.549	0.755	0.744	0.574	0.727	0.743	0.560	0.073	0.043	0.255
	ET	0.722	0.661	0.773	0.711	0.373	0.732	0.905	0.789	0.742	0.796	0.505	0.065	0.031	0.270
	GB	0.713	0.624	0.773	0.716	0.466	0.742	0.810	0.679	0.743	0.760	0.552	0.069	0.038	0.263
	LR	0.717	0.644	0.785	0.696	0.273	0.724	0.876	0.600	0.742	0.775	0.374	0.071	0.034	0.277
	NB	0.622	0.543	0.703	0.589	0.120	0.624	0.855	0.334	0.649	0.697	0.176	0.057	0.043	0.376
	RF	0.686	0.623	0.742	0.669	0.295	0.693	0.889	0.703	0.707	0.763	0.415	0.064	0.035	0.308
	SVC	0.574	0.479	0.674	0.533	0.067	0.574	0.673	0.045	0.606	0.595	0.054	0.070	0.061	0.426
	XGB	0.747	0.683	0.797	0.743	0.408	0.762	0.856	0.696	0.768	0.795	0.513	0.069	0.032	0.240
Algorithm Adaptation Approaches	BRKNNa	0.720	0.656	0.784	0.711	0.357	0.731	0.897	0.705	0.741	0.793	0.473	0.061	0.031	0.270
	BRKNNb	0.009	0.006	0.005	0.012	0.077	0.010	0.012	0.055	0.011	0.012	0.059	0.003	0.164	0.990
	MLKNN	0.741	0.687	0.781	0.746	0.488	0.763	0.835	0.709	0.763	0.783	0.584	0.066	0.032	0.232
	MLARAM	0.574	0.479	0.688	0.533	0.067	0.574	0.673	0.045	0.606	0.595	0.054	0.070	0.061	0.426

Table S6. Performance analysis of data transformation and algorithm adaptation based requirements multilabel classification predictive pipelines using OkapiBm25 representation method and classifiers optimal hyper-parameters over promise dataset

Problem Transformation	Classifier	Accuracy	Subset Accuracy	Precision	Micro Precision	Macro Precision	Recall	Micro Recall	Macro Recall	F1 Score	Micro F1	Macro F1	Average Precision	Hamming Loss	Ranking Loss
Binary Relevance	AB	0.538	0.416	0.598	0.633	0.441	0.592	0.776	0.567	0.577	0.697	0.495	0.062	0.056	0.418
	DT	0.486	0.337	0.544	0.613	0.466	0.581	0.643	0.498	0.535	0.627	0.478	0.064	0.075	0.437
	ET	0.450	0.365	0.511	0.537	0.310	0.472	0.849	0.544	0.477	0.657	0.394	0.050	0.057	0.533
	GB	0.508	0.365	0.572	0.610	0.438	0.579	0.723	0.515	0.553	0.662	0.470	0.065	0.063	0.434
	LR	0.624	0.544	0.662	0.706	0.458	0.659	0.837	0.582	0.648	0.766	0.512	0.067	0.044	0.347
	RF	0.374	0.289	0.442	0.459	0.212	0.385	0.864	0.433	0.400	0.599	0.284	0.045	0.062	0.617
	SVC	0.488	0.422	0.533	0.587	0.322	0.506	0.893	0.558	0.508	0.708	0.407	0.054	0.049	0.497
	XGB	0.391	0.265	0.457	0.505	0.291	0.443	0.678	0.402	0.431	0.579	0.337	0.051	0.075	0.567
Label Powerset	AB	0.396	0.261	0.459	0.529	0.184	0.435	0.445	0.228	0.434	0.483	0.203	0.064	0.116	0.571
	DT	0.453	0.384	0.494	0.532	0.321	0.478	0.562	0.345	0.474	0.546	0.332	0.053	0.090	0.527
	ET	0.646	0.576	0.679	0.723	0.450	0.673	0.701	0.497	0.666	0.711	0.472	0.072	0.060	0.332
	GB	0.602	0.519	0.639	0.683	0.453	0.638	0.654	0.471	0.627	0.668	0.461	0.067	0.069	0.369
	LR	0.735	0.668	0.766	0.793	0.552	0.759	0.778	0.590	0.754	0.785	0.570	0.078	0.044	0.246
	RF	0.606	0.530	0.640	0.693	0.391	0.635	0.662	0.434	0.627	0.677	0.411	0.067	0.068	0.371
	SVC	0.652	0.576	0.679	0.735	0.417	0.682	0.688	0.494	0.672	0.711	0.452	0.072	0.061	0.324
	XGB	0.547	0.466	0.586	0.624	0.381	0.573	0.630	0.411	0.570	0.627	0.395	0.063	0.076	0.432
Classifier Chain	AB	0.568	0.483	0.612	0.642	0.433	0.604	0.786	0.560	0.595	0.706	0.487	0.061	0.054	0.403
	DT	0.489	0.366	0.527	0.601	0.443	0.584	0.624	0.467	0.531	0.612	0.453	0.066	0.078	0.436
	ET	0.462	0.395	0.495	0.566	0.288	0.485	0.827	0.486	0.481	0.671	0.361	0.053	0.056	0.520
	GB	0.487	0.383	0.521	0.580	0.434	0.564	0.650	0.478	0.522	0.612	0.454	0.062	0.075	0.452
	LR	0.640	0.575	0.671	0.716	0.466	0.666	0.851	0.592	0.659	0.777	0.521	0.067	0.042	0.340
	RF	0.372	0.311	0.413	0.454	0.214	0.387	0.856	0.412	0.390	0.592	0.280	0.042	0.063	0.616
	SVC	0.516	0.450	0.544	0.622	0.336	0.539	0.863	0.552	0.534	0.722	0.417	0.057	0.049	0.466
	XGB	0.422	0.335	0.462	0.523	0.278	0.455	0.681	0.397	0.447	0.590	0.326	0.052	0.075	0.551
Algorithm Adaptation Approaches	BRKNNa	0.412	0.317	0.496	0.519	0.253	0.449	0.566	0.372	0.437	0.541	0.302	0.049	0.080	0.427
	BRKNNb	0.071	0.027	0.088	0.110	0.119	0.106	0.100	0.071	0.092	0.105	0.086	0.013	0.189	0.589
	MLKNN	0.457	0.335	0.485	0.546	0.351	0.510	0.692	0.461	0.494	0.608	0.397	0.057	0.071	0.498
	MLARAM	0.404	0.281	0.621	0.539	0.225	0.454	0.442	0.223	0.438	0.486	0.216	0.061	0.103	0.532

Table S7. Performance analysis of data transformation and algorithm adaptation based requirements multilabel classification predictive pipelines using Word2vec pre-trained embeddings over 9 different classifiers across EHR-Binary dataset

Problem Transformation	Classifier	Accuracy	Subset Accuracy	Precision	Micro Precision	Macro Precision	Recall	Micro Recall	Macro Recall	F1 Score	Micro F1	Macro F1	Average Precision	Hamming Loss	Ranking Loss
Binary Relevance	AB	0.737	0.606	0.779	0.815	0.806	0.826	0.789	0.782	0.781	0.802	0.794	0.585	0.240	0.306
	DT	0.641	0.496	0.682	0.739	0.731	0.744	0.743	0.735	0.689	0.741	0.733	0.559	0.307	0.409
	ET	0.766	0.623	0.801	0.863	0.853	0.874	0.788	0.783	0.814	0.824	0.816	0.598	0.219	0.305
	GB	0.804	0.685	0.838	0.877	0.870	0.889	0.816	0.811	0.844	0.846	0.839	0.606	0.191	0.246
	LR	0.757	0.566	0.792	0.897	0.887	0.913	0.747	0.744	0.821	0.815	0.809	0.622	0.241	0.364
	NB	0.662	0.510	0.704	0.767	0.759	0.772	0.742	0.735	0.713	0.754	0.747	0.573	0.297	0.398
	RF	0.766	0.629	0.802	0.854	0.845	0.866	0.793	0.786	0.811	0.822	0.814	0.595	0.220	0.295
	SVC	0.818	0.706	0.852	0.884	0.878	0.897	0.825	0.820	0.856	0.854	0.848	0.610	0.180	0.227
	XGB	0.828	0.732	0.859	0.883	0.877	0.894	0.849	0.843	0.860	0.866	0.860	0.597	0.163	0.205
Classifier Chain	AB	0.761	0.674	0.813	0.786	0.773	0.797	0.808	0.800	0.790	0.797	0.786	0.562	0.239	0.223
	DT	0.695	0.587	0.747	0.745	0.739	0.750	0.742	0.735	0.731	0.744	0.737	0.598	0.305	0.308
	ET	0.802	0.726	0.847	0.819	0.798	0.831	0.843	0.851	0.827	0.831	0.823	0.521	0.199	0.182
	GB	0.797	0.727	0.847	0.804	0.787	0.817	0.847	0.846	0.821	0.825	0.815	0.534	0.203	0.173
	LR	0.699	0.612	0.780	0.685	0.644	0.707	0.782	0.816	0.729	0.730	0.720	0.431	0.301	0.228
	NB	0.695	0.561	0.751	0.762	0.754	0.773	0.735	0.728	0.740	0.748	0.740	0.601	0.305	0.327
	RF	0.813	0.725	0.854	0.848	0.832	0.860	0.839	0.841	0.842	0.843	0.836	0.552	0.187	0.193
	SVC	0.789	0.707	0.867	0.760	0.735	0.793	0.868	0.872	0.816	0.811	0.798	0.494	0.211	0.137
	XGB	0.835	0.774	0.877	0.842	0.830	0.854	0.876	0.874	0.856	0.859	0.851	0.555	0.165	0.142
Label Powerset	AB	0.755	0.668	0.813	0.769	0.757	0.784	0.809	0.800	0.784	0.789	0.778	0.563	0.245	0.216
	DT	0.703	0.592	0.757	0.751	0.743	0.759	0.749	0.742	0.739	0.750	0.742	0.594	0.297	0.299
	ET	0.818	0.752	0.876	0.804	0.788	0.826	0.879	0.880	0.840	0.840	0.831	0.528	0.182	0.132
	GB	0.815	0.750	0.863	0.819	0.810	0.833	0.863	0.858	0.837	0.841	0.833	0.564	0.185	0.155
	LR	0.775	0.692	0.847	0.757	0.738	0.784	0.847	0.842	0.802	0.800	0.787	0.520	0.226	0.162
	NB	0.705	0.555	0.743	0.814	0.809	0.817	0.724	0.719	0.755	0.766	0.761	0.639	0.295	0.369
	RF	0.816	0.750	0.872	0.806	0.791	0.825	0.875	0.873	0.838	0.839	0.830	0.537	0.184	0.138
	SVC	0.815	0.746	0.872	0.805	0.794	0.826	0.873	0.868	0.838	0.838	0.829	0.552	0.185	0.139
	XGB	0.846	0.788	0.887	0.851	0.843	0.863	0.886	0.881	0.865	0.868	0.862	0.574	0.154	0.130
Algorithm Adaptation Approaches	BRKNNa	0.540	0.442	0.623	0.540	0.507	0.546	0.635	0.594	0.573	0.584	0.547	0.459	0.458	0.373
	BRKNNb	0.541	0.447	0.624	0.535	0.502	0.541	0.635	0.595	0.572	0.580	0.544	0.459	0.459	0.366
	MLARAM	0.576	0.482	0.670	0.564	0.500	0.576	0.670	0.385	0.607	0.613	0.424	0.335	0.424	0.330
	MLKNN	0.587	0.261	0.594	0.881	0.864	0.885	0.605	0.593	0.695	0.717	0.702	0.702	0.413	0.685

Table S8. Performance analysis of data transformation and algorithm adaptation based requirements multilabel classification predictive pipelines using Word2vec pre-trained embeddings over 9 different classifiers across EHR-Multiclass dataset

Problem Transformation	Classifier	Accuracy	Subset Accuracy	Precision	Micro Precision	Macro Precision	Recall	Micro Recall	Macro Recall	F1 Score	Micro F1	Macro F1	Average Precision	Hamming Loss	Ranking Loss
Binary Relevance	AB	0.621	0.510	0.684	0.645	0.328	0.672	0.761	0.523	0.659	0.698	0.402	0.066	0.047	0.336
	DT	0.510	0.373	0.557	0.599	0.298	0.618	0.559	0.264	0.560	0.578	0.280	0.072	0.074	0.403
	ET	0.601	0.527	0.672	0.575	0.140	0.607	0.830	0.421	0.626	0.679	0.210	0.062	0.046	0.394
	GB	0.645	0.552	0.710	0.646	0.260	0.677	0.805	0.495	0.677	0.716	0.341	0.066	0.043	0.329
	LR	0.559	0.469	0.654	0.517	0.068	0.559	0.764	0.169	0.590	0.617	0.094	0.059	0.054	0.441
	NB	0.502	0.326	0.538	0.673	0.436	0.686	0.442	0.232	0.568	0.534	0.303	0.091	0.099	0.356
	RF	0.600	0.520	0.672	0.581	0.156	0.612	0.827	0.487	0.628	0.682	0.236	0.060	0.046	0.390
	SVC	0.607	0.528	0.683	0.576	0.130	0.612	0.867	0.323	0.634	0.692	0.185	0.057	0.043	0.389
	XGB	0.692	0.618	0.750	0.682	0.295	0.709	0.876	0.692	0.717	0.767	0.413	0.063	0.035	0.294
Classifier Chain	AB	0.639	0.539	0.703	0.650	0.325	0.676	0.752	0.552	0.673	0.697	0.408	0.064	0.048	0.330
	DT	0.524	0.405	0.575	0.582	0.281	0.604	0.544	0.247	0.566	0.562	0.262	0.067	0.077	0.411
	ET	0.640	0.566	0.714	0.609	0.166	0.643	0.837	0.517	0.665	0.705	0.251	0.063	0.043	0.357
	GB	0.661	0.574	0.730	0.652	0.255	0.684	0.794	0.473	0.691	0.716	0.331	0.065	0.044	0.320
	LR	0.596	0.500	0.696	0.553	0.096	0.596	0.740	0.217	0.628	0.633	0.133	0.064	0.054	0.404
	NB	0.517	0.352	0.552	0.667	0.425	0.679	0.439	0.239	0.577	0.529	0.306	0.082	0.101	0.357
	RF	0.631	0.556	0.705	0.602	0.160	0.636	0.843	0.494	0.657	0.702	0.241	0.063	0.043	0.365
	SVC	0.644	0.546	0.747	0.595	0.189	0.645	0.748	0.265	0.678	0.663	0.220	0.063	0.051	0.355
	XGB	0.703	0.632	0.762	0.690	0.299	0.716	0.859	0.650	0.727	0.765	0.409	0.065	0.036	0.286
Label Powerset	AB	0.572	0.478	0.672	0.530	0.067	0.573	0.672	0.047	0.605	0.593	0.055	0.070	0.062	0.428
	DT	0.529	0.438	0.575	0.554	0.226	0.577	0.549	0.223	0.560	0.552	0.224	0.065	0.076	0.431
	ET	0.663	0.589	0.735	0.636	0.185	0.668	0.747	0.585	0.688	0.687	0.281	0.074	0.049	0.333
	GB	0.662	0.581	0.718	0.663	0.270	0.688	0.693	0.365	0.689	0.678	0.310	0.074	0.053	0.316
	LR	0.594	0.504	0.683	0.561	0.088	0.599	0.685	0.198	0.625	0.617	0.121	0.071	0.059	0.402
	NB	0.577	0.471	0.621	0.628	0.327	0.639	0.607	0.347	0.613	0.617	0.336	0.065	0.066	0.371
	RF	0.665	0.591	0.734	0.641	0.186	0.673	0.746	0.533	0.690	0.689	0.275	0.074	0.049	0.329
	SVC	0.669	0.590	0.741	0.645	0.174	0.678	0.751	0.431	0.695	0.694	0.248	0.075	0.048	0.324
	XGB	0.687	0.606	0.747	0.680	0.317	0.710	0.741	0.516	0.714	0.720	0.393	0.053	0.024	0.254
Algorithm Adaptation Approaches	BRKNNa	0.398	0.331	0.513	0.369	0.057	0.399	0.560	0.065	0.421	0.443	0.059	0.050	0.077	0.600
	BRKNNb	0.007	0.005	0.006	0.008	0.071	0.008	0.010	0.001	0.008	0.009	0.002	0.002	0.155	0.991
	MLARAM	0.483	0.383	0.026	0.486	0.08	0.524	0.566	0.041	0.522	0.512	0.051	0.081	0.082	0.485
	MLKNN	0.570	0.475	0.670	0.528	0.066	0.570	0.673	0.044	0.602	0.591	0.053	0.069	0.061	0.429

Table S9. Performance analysis of data transformation and algorithm adaptation based requirements multilabel classification predictive pipelines using Word2vec pre-trained embeddings over 9 different classifiers across promise dataset

Problem Transformation	Classifier	Accuracy	Subset Accuracy	Precision	Micro Precision	Macro Precision	Recall	Micro Recall	Macro Recall	F1 Score	Micro F1	Macro F1	Average Precision	Hamming Loss	Ranking Loss
Binary Relevance	AB	0.395	0.255	0.473	0.505	0.282	0.443	0.672	0.393	0.438	0.576	0.328	0.050	0.076	0.566
	DT	0.290	0.120	0.372	0.453	0.252	0.397	0.435	0.231	0.349	0.443	0.240	0.053	0.116	0.628
	ET	0.222	0.136	0.313	0.299	0.101	0.225	0.767	0.282	0.251	0.429	0.148	0.032	0.081	0.776
	GB	0.345	0.234	0.417	0.443	0.228	0.371	0.731	0.410	0.378	0.550	0.292	0.043	0.073	0.633
	LR	0.035	0.004	0.085	0.053	0.012	0.035	0.861	0.054	0.049	0.099	0.020	0.005	0.097	0.965
	NB	0.356	0.236	0.403	0.500	0.281	0.443	0.513	0.294	0.396	0.506	0.286	0.058	0.099	0.581
	RF	0.241	0.145	0.337	0.320	0.111	0.249	0.736	0.285	0.273	0.444	0.158	0.031	0.081	0.752
	SVC	0.202	0.109	0.286	0.286	0.076	0.204	0.853	0.164	0.229	0.427	0.104	0.027	0.078	0.796
	XGB	0.387	0.299	0.454	0.480	0.217	0.400	0.840	0.454	0.413	0.610	0.293	0.044	0.062	0.603
Classifier Chain	AB	0.432	0.346	0.470	0.531	0.283	0.465	0.662	0.379	0.457	0.589	0.323	0.049	0.075	0.541
	DT	0.290	0.145	0.330	0.455	0.249	0.402	0.438	0.252	0.338	0.445	0.249	0.053	0.116	0.625
	ET	0.285	0.225	0.320	0.392	0.141	0.292	0.864	0.341	0.300	0.538	0.198	0.035	0.068	0.709
	GB	0.371	0.283	0.401	0.498	0.251	0.409	0.713	0.405	0.395	0.586	0.309	0.047	0.072	0.598
	LR	0.085	0.073	0.087	0.143	0.046	0.091	0.783	0.146	0.088	0.241	0.069	0.012	0.091	0.910
	NB	0.384	0.265	0.412	0.537	0.297	0.474	0.495	0.278	0.419	0.515	0.287	0.057	0.103	0.549
	RF	0.265	0.198	0.311	0.363	0.121	0.270	0.885	0.306	0.282	0.513	0.172	0.033	0.070	0.731
	SVC	0.306	0.215	0.350	0.412	0.154	0.327	0.468	0.150	0.330	0.438	0.151	0.043	0.108	0.677
	XGB	0.414	0.341	0.451	0.529	0.244	0.435	0.801	0.446	0.434	0.636	0.314	0.048	0.061	0.569
Label Powerset	AB	0.299	0.178	0.338	0.458	0.147	0.347	0.365	0.074	0.331	0.401	0.096	0.052	0.137	0.662
	DT	0.298	0.218	0.335	0.374	0.205	0.331	0.382	0.207	0.322	0.378	0.205	0.040	0.126	0.676
	ET	0.565	0.479	0.603	0.653	0.360	0.597	0.623	0.418	0.590	0.637	0.387	0.066	0.076	0.409
	GB	0.437	0.349	0.471	0.544	0.291	0.478	0.508	0.294	0.463	0.525	0.292	0.057	0.100	0.531
	LR	0.384	0.269	0.420	0.575	0.188	0.437	0.435	0.139	0.417	0.495	0.159	0.067	0.120	0.573
	NB	0.432	0.354	0.471	0.519	0.256	0.456	0.529	0.272	0.453	0.524	0.264	0.054	0.097	0.549
	RF	0.557	0.481	0.589	0.661	0.324	0.589	0.612	0.368	0.579	0.635	0.344	0.070	0.078	0.417
	SVC	0.475	0.381	0.508	0.607	0.242	0.514	0.539	0.204	0.501	0.571	0.221	0.067	0.094	0.493
	XGB	0.536	0.453	0.581	0.601	0.370	0.564	0.619	0.396	0.561	0.609	0.382	0.059	0.079	0.442
Algorithm Adptation Approaches	BRKNNa	BRKNNa	0.068	0.029	0.025	0.095	0.030	0.079	0.342	0.050	0.082	0.118	0.031	0.050	0.109
	BRKNNb	BRKNNb	0.059	0.016	0.057	0.070	0.104	0.099	0.065	0.010	0.075	0.067	0.018	0.049	0.191
	MLARAM	MLARAM	0.306	0.146	0.379	0.473	0.129	0.352	0.376	0.050	0.349	0.418	0.072	0.05	0.135
	MLKNN	MLKNN	0.009	0.001	0.012	0.014	0.004	0.009	0.315	0.021	0.013	0.026	0.005	0.050	0.102

Table S10. Performance analysis of data transformation and algorithm adaptation based requirements multilabel classification predictive pipelines using Fasttext pretrained embedddings over 9 different classifiers across EHR-Binary dataset

Problem Transformation	Classifier	Accuracy	Subset Accuracy	Precision	Micro Precision	Macro Precision	Recall	Micro Recall	Macro Recall	F1 Score	Micro F1	Macro F1	Average Precision	Hamming Loss	Ranking Loss
Binary Relevance	AB	0.765	0.640	0.810	0.830	0.822	0.846	0.812	0.805	0.807	0.821	0.813	0.586	0.215	0.268
	DT	0.657	0.516	0.697	0.748	0.739	0.757	0.756	0.747	0.704	0.752	0.743	0.557	0.293	0.388
	ET	0.821	0.695	0.846	0.912	0.905	0.922	0.812	0.809	0.863	0.859	0.854	0.611	0.178	0.254
	GB	0.816	0.703	0.853	0.877	0.869	0.892	0.832	0.826	0.853	0.854	0.847	0.599	0.178	0.223
	LR	0.810	0.690	0.853	0.868	0.859	0.887	0.827	0.820	0.850	0.847	0.839	0.597	0.187	0.224
	NB	0.722	0.600	0.781	0.764	0.754	0.784	0.788	0.779	0.762	0.775	0.766	0.573	0.263	0.276
	RF	0.817	0.691	0.845	0.905	0.898	0.915	0.814	0.810	0.859	0.857	0.851	0.611	0.180	0.253
	SVC	0.849	0.759	0.883	0.890	0.883	0.904	0.865	0.860	0.879	0.877	0.871	0.594	0.148	0.172
	XGB	0.845	0.756	0.876	0.891	0.884	0.903	0.864	0.859	0.874	0.877	0.871	0.593	0.149	0.181
Classifier Chain	AB	0.786	0.698	0.838	0.807	0.795	0.823	0.829	0.821	0.816	0.818	0.808	0.567	0.214	0.198
	DT	0.713	0.604	0.768	0.758	0.750	0.767	0.759	0.751	0.749	0.758	0.751	0.595	0.287	0.286
	ET	0.814	0.743	0.863	0.823	0.802	0.838	0.859	0.870	0.838	0.840	0.835	0.519	0.186	0.161
	GB	0.819	0.751	0.869	0.818	0.802	0.835	0.869	0.869	0.841	0.843	0.834	0.535	0.182	0.147
	LR	0.811	0.739	0.868	0.805	0.786	0.826	0.867	0.869	0.835	0.835	0.825	0.525	0.189	0.146
	NB	0.740	0.628	0.809	0.759	0.749	0.783	0.794	0.785	0.777	0.776	0.766	0.577	0.260	0.234
	RF	0.827	0.741	0.872	0.851	0.836	0.868	0.857	0.858	0.856	0.854	0.847	0.551	0.173	0.170
	SVC	0.842	0.778	0.897	0.830	0.814	0.853	0.897	0.897	0.864	0.862	0.854	0.534	0.158	0.114
	XGB	0.846	0.784	0.888	0.851	0.838	0.865	0.885	0.883	0.866	0.867	0.860	0.555	0.154	0.131
Label Powerset	AB	0.766	0.677	0.822	0.783	0.772	0.798	0.815	0.806	0.795	0.799	0.788	0.571	0.234	0.211
	DT	0.710	0.599	0.764	0.760	0.752	0.768	0.755	0.747	0.747	0.757	0.749	0.597	0.290	0.294
	ET	0.824	0.758	0.887	0.803	0.786	0.828	0.891	0.893	0.846	0.845	0.836	0.525	0.176	0.117
	GB	0.828	0.762	0.879	0.825	0.816	0.843	0.878	0.873	0.850	0.851	0.843	0.561	0.172	0.137
	LR	0.824	0.755	0.878	0.820	0.809	0.839	0.876	0.871	0.847	0.847	0.839	0.559	0.176	0.138
	NB	0.751	0.641	0.788	0.820	0.816	0.823	0.775	0.770	0.788	0.797	0.792	0.629	0.249	0.284
	RF	0.823	0.756	0.885	0.802	0.788	0.828	0.889	0.888	0.845	0.843	0.835	0.532	0.177	0.119
	SVC	0.851	0.790	0.901	0.843	0.833	0.863	0.900	0.896	0.871	0.870	0.863	0.559	0.149	0.111
	XGB	0.856	0.798	0.899	0.853	0.845	0.869	0.898	0.895	0.875	0.875	0.869	0.566	0.145	0.115
Algorithm Adaptation Techniques	BRKNNa	0.7827	0.701	0.807	0.8209	0.8139	0.8299	0.814	0.808	0.8099	0.8174	0.8109	0.549	0.22	0.2294
	BRKNNb	0.7867	0.72	0.807	0.8059	0.802	0.8152	0.82	0.8147	0.8089	0.8128	0.8083	0.5469	0.2133	0.2036
	MLARAM	0.5761	0.4822	0.6696	0.5641	0.5002	0.5761	0.6702	0.3851	0.6074	0.6126	0.4237	0.335	0.4239	0.33
	MLKNN	0.7927	0.711	0.817	0.8309	0.8239	0.8399	0.824	0.818	0.8199	0.8274	0.8209	0.559	0.2	0.2194

Table S11. Performance analysis of data transformation and algorithm adaptation based requirements multilabel classification predictive pipelines using Fasttext pre-trained embedddings over 9 different classifiers across EHR-Multiclass dataset

Problem Transformation	Classifier	Accuracy	Subset Accuracy	Precision	Micro Precision	Macro Precision	Recall	Micro Recall	Macro Recall	F1 Score	Micro F1	Macro F1	Average Precision	AUROC	Micro AUROC	Macro AUROC	Hamming Loss	Ranking Loss
Binary Relevance	AB	0.630	0.522	0.690	0.652	0.342	0.680	0.766	0.543	0.667	0.704	0.418	0.067	0.742	0.817	0.655	0.046	0.328
	DT	0.523	0.388	0.572	0.604	0.288	0.623	0.572	0.263	0.572	0.588	0.275	0.073	0.684	0.781	0.615	0.072	0.395
	ET	0.625	0.550	0.699	0.595	0.152	0.629	0.840	0.498	0.650	0.696	0.232	0.060	0.688	0.792	0.562	0.044	0.372
	GB	0.648	0.555	0.714	0.646	0.262	0.678	0.812	0.511	0.680	0.720	0.346	0.067	0.743	0.816	0.618	0.043	0.327
	LR	0.626	0.546	0.699	0.600	0.156	0.635	0.861	0.372	0.653	0.707	0.219	0.059	0.720	0.796	0.567	0.042	0.366
	NB	0.534	0.348	0.565	0.724	0.555	0.739	0.480	0.270	0.605	0.577	0.363	0.095	0.757	0.826	0.735	0.090	0.304
	RF	0.618	0.545	0.690	0.590	0.152	0.623	0.845	0.487	0.643	0.695	0.232	0.060	0.692	0.790	0.563	0.044	0.378
	SVC	0.674	0.601	0.739	0.653	0.216	0.684	0.890	0.477	0.699	0.753	0.297	0.061	0.765	0.823	0.599	0.036	0.318
	XGB	0.690	0.616	0.750	0.680	0.289	0.706	0.880	0.690	0.715	0.767	0.407	0.064	0.775	0.836	0.635	0.035	0.296
Classifier Chain	AB	0.640	0.542	0.705	0.651	0.317	0.676	0.761	0.507	0.674	0.701	0.389	0.064	0.739	0.816	0.643	0.047	0.329
	DT	0.533	0.412	0.584	0.597	0.277	0.617	0.567	0.256	0.577	0.582	0.266	0.071	0.681	0.778	0.609	0.073	0.398
	ET	0.642	0.565	0.719	0.609	0.168	0.644	0.852	0.515	0.668	0.710	0.253	0.064	0.701	0.799	0.571	0.042	0.357
	GB	0.661	0.574	0.728	0.653	0.261	0.684	0.801	0.501	0.691	0.720	0.343	0.067	0.741	0.819	0.616	0.043	0.319
	LR	0.668	0.582	0.748	0.640	0.211	0.678	0.824	0.478	0.697	0.721	0.292	0.066	0.730	0.814	0.592	0.042	0.324
	NB	0.543	0.369	0.575	0.716	0.561	0.731	0.478	0.271	0.610	0.573	0.365	0.086	0.760	0.822	0.739	0.090	0.309
	RF	0.630	0.556	0.704	0.597	0.155	0.633	0.857	0.443	0.655	0.704	0.230	0.061	0.702	0.794	0.565	0.043	0.368
	SVC	0.709	0.630	0.782	0.683	0.279	0.717	0.792	0.438	0.736	0.733	0.340	0.069	0.769	0.833	0.626	0.042	0.284
	XGB	0.705	0.637	0.765	0.690	0.299	0.715	0.872	0.676	0.728	0.770	0.414	0.067	0.776	0.840	0.639	0.035	0.286
Label Powerset	AB	0.573	0.478	0.673	0.531	0.067	0.573	0.673	0.045	0.606	0.593	0.054	0.070	0.500	0.754	0.500	0.062	0.427
	DT	0.535	0.441	0.584	0.561	0.229	0.584	0.558	0.223	0.568	0.559	0.226	0.065	0.654	0.760	0.585	0.075	0.424
	ET	0.674	0.600	0.749	0.642	0.198	0.677	0.761	0.591	0.699	0.697	0.296	0.074	0.626	0.812	0.574	0.047	0.323
	GB	0.650	0.568	0.709	0.654	0.263	0.679	0.678	0.329	0.679	0.666	0.292	0.073	0.688	0.813	0.606	0.056	0.326
	LR	0.703	0.622	0.774	0.680	0.233	0.714	0.778	0.473	0.730	0.726	0.311	0.075	0.694	0.831	0.596	0.044	0.288
	NB	0.605	0.500	0.643	0.667	0.436	0.676	0.630	0.372	0.641	0.648	0.401	0.067	0.761	0.815	0.695	0.061	0.335
	RF	0.674	0.598	0.748	0.644	0.192	0.678	0.759	0.518	0.700	0.697	0.279	0.074	0.630	0.813	0.571	0.047	0.323
	SVC	0.739	0.668	0.804	0.719	0.311	0.750	0.812	0.614	0.764	0.763	0.412	0.078	0.730	0.852	0.637	0.038	0.252
	XGB	0.702	0.622	0.758	0.700	0.352	0.728	0.754	0.555	0.729	0.726	0.430	0.073	0.734	0.839	0.657	0.045	0.276
Algorithm Adaptation Approaches	BRKNNa	0.654	0.582	0.716	0.648	0.247	0.671	0.857	0.564	0.678	0.738	0.344	0.059	0.759	0.819	0.613	0.038	0.331
	BRKNNb	0.008	0.005	0.007	0.01	0.076	0.009	0.009	0.014	0.009	0.009	0.016	0.002	0.499	0.458	0.495	0.169	0.990
	MLARAM	0.483	0.383	0.026	0.486	0.08	0.524	0.566	0.041	0.522	0.512	0.051	0.081	0.5	0.721	0.5	0.082	0.485
	MLKNN	0.694	0.608	0.753	0.704	0.389	0.726	0.820	0.632	0.723	0.758	0.480	0.065	0.785	0.845	0.682	0.038	0.278

Table S12. Performance analysis of data transformation and algorithm adaptation based requirements multilabel classification predictive pipelines using Fasttext pretrained embedddings over 9 different classifiers across promise dataset

Problem Transformation	Classifier	Accuracy	Subset Accuracy	Precision	Micro Precision	Macro Precision	Recall	Micro Recall	Macro Recall	F1 Score	Micro F1	Macro F1	Average Precision	Hamming Loss	Ranking Loss
Binary Relevance	AB	0.405	0.279	0.475	0.506	0.300	0.459	0.666	0.413	0.446	0.575	0.347	0.051	0.076	0.550
	DT	0.289	0.121	0.364	0.447	0.258	0.400	0.432	0.236	0.350	0.439	0.245	0.053	0.117	0.625
	ET	0.250	0.164	0.337	0.327	0.114	0.251	0.893	0.333	0.277	0.476	0.169	0.030	0.073	0.749
	GB	0.368	0.268	0.437	0.465	0.216	0.395	0.732	0.394	0.399	0.568	0.278	0.047	0.072	0.610
	LR	0.221	0.136	0.296	0.320	0.092	0.224	0.860	0.191	0.246	0.465	0.123	0.031	0.075	0.777
	NB	0.420	0.231	0.460	0.632	0.433	0.586	0.503	0.345	0.482	0.560	0.383	0.067	0.102	0.450
	RF	0.260	0.184	0.335	0.334	0.118	0.263	0.858	0.349	0.284	0.479	0.175	0.032	0.074	0.738
	SVC	0.345	0.265	0.405	0.442	0.157	0.349	0.872	0.334	0.366	0.586	0.212	0.040	0.063	0.652
	XGB	0.420	0.335	0.485	0.508	0.242	0.432	0.836	0.454	0.445	0.631	0.315	0.048	0.061	0.571
Classifier Chain	AB	0.454	0.360	0.492	0.545	0.335	0.501	0.661	0.422	0.482	0.597	0.373	0.052	0.075	0.508
	DT	0.303	0.165	0.337	0.449	0.272	0.408	0.439	0.247	0.347	0.443	0.257	0.050	0.114	0.616
	ET	0.310	0.250	0.344	0.419	0.153	0.319	0.839	0.346	0.325	0.558	0.211	0.038	0.068	0.683
	GB	0.406	0.308	0.431	0.548	0.253	0.454	0.724	0.390	0.433	0.624	0.306	0.053	0.068	0.555
	LR	0.351	0.280	0.375	0.479	0.187	0.374	0.754	0.298	0.368	0.584	0.229	0.044	0.069	0.630
	NB	0.430	0.255	0.454	0.634	0.420	0.581	0.490	0.337	0.484	0.552	0.373	0.066	0.105	0.451
	RF	0.287	0.227	0.328	0.384	0.138	0.291	0.847	0.356	0.303	0.526	0.198	0.035	0.070	0.710
	SVC	0.499	0.414	0.538	0.599	0.281	0.528	0.629	0.292	0.522	0.613	0.286	0.057	0.077	0.477
	XGB	0.428	0.356	0.455	0.547	0.257	0.457	0.769	0.441	0.448	0.639	0.324	0.051	0.063	0.549
Label Powerset	AB	0.316	0.173	0.373	0.496	0.138	0.363	0.386	0.073	0.355	0.434	0.095	0.062	0.133	0.645
	DT	0.326	0.242	0.368	0.409	0.236	0.360	0.412	0.236	0.352	0.410	0.235	0.043	0.120	0.647
	ET	0.569	0.492	0.600	0.670	0.353	0.603	0.619	0.408	0.591	0.643	0.378	0.068	0.076	0.404
	GB	0.463	0.365	0.504	0.568	0.283	0.508	0.525	0.274	0.492	0.545	0.278	0.061	0.097	0.501
	LR	0.533	0.450	0.561	0.646	0.291	0.568	0.582	0.286	0.556	0.612	0.287	0.068	0.084	0.438
	NB	0.516	0.423	0.558	0.612	0.360	0.546	0.602	0.376	0.541	0.607	0.367	0.059	0.081	0.460
	RF	0.559	0.476	0.592	0.663	0.344	0.591	0.613	0.388	0.582	0.637	0.364	0.067	0.078	0.415
	SVC	0.600	0.523	0.638	0.685	0.343	0.628	0.659	0.375	0.622	0.671	0.357	0.071	0.068	0.377
	XGB	0.583	0.501	0.622	0.662	0.420	0.611	0.649	0.460	0.606	0.655	0.438	0.063	0.072	0.395
Algorithm Adaptation Techniques	BRKNNa	0.318	0.233	0.390	0.433	0.162	0.335	0.762	0.311	0.342	0.552	0.212	0.041	0.071	0.667
	BRKNNb	0.066	0.021	0.065	0.097	0.111	0.101	0.091	0.058	0.086	0.094	0.076	0.011	0.190	0.905
	MLARAM	0.306	0.146	0.379	0.473	0.129	0.352	0.376	0.050	0.349	0.418	0.072	0.061	0.135	0.656
	MLKNN	0.418	0.315	0.497	0.513	0.288	0.448	0.728	0.457	0.448	0.601	0.352	0.050	0.069	0.556

Table S13. Performance comparison of 9 adapted deep learning predictors based on 3 different embeddings over promise dataset

**Embedding**	**Metric**	**DL Predictor**								
		Region Embedding	FastText	Text CNN	DPCNN	Text VDCNN	Attentive ConvNet	Text RNN	DRNN	Text RCNN
Random Embedding	Precision	0.589	0.597	0.634	0.557	0.363	0.314	0.395	0.529	0.611
	Avg_Precision	0.224	0.230	0.237	0.231	0.245	0.092	0.246	0.254	0.244
	Micro_Precision	0.618	0.647	0.678	0.612	0.421	0.258	0.479	0.608	0.662
	Macro_Precision	0.361	0.360	0.426	0.363	0.126	0.119	0.162	0.301	0.402
	Recall	0.565	0.584	0.630	0.550	0.325	0.225	0.361	0.537	0.608
	Micro_Recall	0.825	0.852	0.796	0.732	0.374	0.534	0.607	0.695	0.714
	Macro_Recall	0.497	0.507	0.535	0.432	0.062	0.221	0.174	0.363	0.453
	F1-score	0.566	0.581	0.620	0.538	0.314	0.243	0.364	0.519	0.595
	Micro_F1	0.705	0.734	0.731	0.666	0.377	0.338	0.529	0.644	0.686
	Macro_F1	0.416	0.419	0.473	0.394	0.081	0.152	0.162	0.328	0.425
	Acuuracy	0.540	0.558	0.592	0.508	0.263	0.203	0.330	0.490	0.562
	Subset Accuracy	0.453	0.481	0.497	0.405	0.104	0.093	0.213	0.385	0.452
	Hamming Loss	0.051	0.047	0.050	0.062	0.137	0.103	0.088	0.067	0.061
	Ranking Loss	0.440	0.421	0.376	0.457	0.690	0.780	0.644	0.473	0.400
FastText	Precision	0.622	0.612	0.668	0.594	0.323	0.328	0.396	0.511	0.651
	Avg_Precision	0.229	0.231	0.231	0.239	0.260	0.105	0.158	0.241	0.251
	Micro_Precision	0.656	0.651	0.691	0.645	0.525	0.294	0.378	0.584	0.702
	Macro_Precision	0.409	0.368	0.454	0.374	0.240	0.141	0.127	0.297	0.438
	Recall	0.600	0.590	0.651	0.586	0.435	0.264	0.297	0.515	0.658
	Micro_Recall	0.847	0.852	0.825	0.741	0.341	0.777	0.505	0.698	0.743
	Macro_Recall	0.555	0.526	0.572	0.466	0.080	0.287	0.114	0.385	0.478
	F1-score	0.601	0.590	0.645	0.577	0.295	0.279	0.321	0.499	0.642
	Micro_F1	0.738	0.737	0.751	0.688	0.354	0.396	0.423	0.634	0.721
	Macro_F1	0.470	0.431	0.503	0.413	0.108	0.184	0.113	0.333	0.456
	Acuuracy	0.578	0.567	0.618	0.548	0.232	0.250	0.279	0.469	0.614
	Subset Accuracy	0.496	0.492	0.530	0.445	0.073	0.161	0.153	0.366	0.518
	Hamming Loss	0.047	0.047	0.046	0.059	0.230	0.080	0.102	0.068	0.055
	Ranking Loss	0.404	0.415	0.355	0.421	0.694	0.739	0.706	0.494	0.350
Word2Vec	Precision	0.628	0.614	0.627	0.558	0.329	0.444	0.449	0.521	0.636
	Avg_Precision	0.233	0.231	0.235	0.229	0.294	0.144	0.223	0.240	0.255
	Micro_Precision	0.657	0.654	0.663	0.608	0.452	0.412	0.512	0.604	0.691
	Macro_Precision	0.396	0.387	0.412	0.364	0.155	0.177	0.223	0.322	0.438
	Recall	0.602	0.597	0.614	0.547	0.348	0.355	0.420	0.540	0.650
	Micro_Recall	0.853	0.844	0.798	0.740	0.360	0.839	0.653	0.678	0.717
	Macro_Recall	0.542	0.531	0.538	0.454	0.063	0.370	0.256	0.374	0.460
	F1-score	0.604	0.595	0.607	0.539	0.308	0.380	0.421	0.512	0.628
	Micro_F1	0.741	0.735	0.723	0.665	0.379	0.548	0.572	0.637	0.704
	Macro_F1	0.457	0.446	0.464	0.403	0.084	0.237	0.237	0.344	0.448
	Acuuracy	0.580	0.572	0.578	0.508	0.257	0.347	0.391	0.481	0.593
	Subset Accuracy	0.501	0.493	0.484	0.408	0.100	0.242	0.287	0.384	0.477
	Hamming Loss	0.046	0.047	0.051	0.061	0.158	0.068	0.077	0.070	0.058
	Ranking Loss	0.402	0.408	0.392	0.460	0.684	0.646	0.586	0.473	0.361

Table S14. Performance comparison of 9 adapted deep learning predictors based on 3 different embeddings over EHR-binary dataset

Embedding	Metric	DL Predictor								
		Region Embedding	FastText	Text CNN	DPCNN	Text VDCNN	Attentive ConvNet	Text RNN	DRNN	Text RCNN
Random Embedding	Precision	0.886	0.903	0.893	0.903	0.883	0.696	0.829	0.893	0.907
	Avg_Precision	0.892	0.904	0.899	0.909	0.889	0.765	0.920	0.882	0.894
	Micro_Precision	0.886	0.899	0.893	0.904	0.883	0.762	0.915	0.876	0.888
	Macro_Precision	0.903	0.917	0.908	0.921	0.906	0.768	0.934	0.896	0.907
	Recall	0.887	0.889	0.882	0.884	0.857	0.758	0.806	0.885	0.895
	Micro_Recall	0.882	0.884	0.878	0.880	0.851	0.770	0.802	0.881	0.891
	Macro_Recall	0.881	0.898	0.889	0.900	0.878	0.707	0.854	0.882	0.896
	F1-score	0.889	0.896	0.890	0.896	0.872	0.744	0.852	0.883	0.894
	Micro_F1	0.884	0.891	0.885	0.891	0.866	0.748	0.847	0.878	0.890
	Macro_F1	0.856	0.873	0.865	0.875	0.845	0.658	0.800	0.858	0.874
	Acuuracy	0.780	0.799	0.793	0.799	0.745	0.512	0.638	0.784	0.809
	Subset Accuracy	0.585	0.597	0.595	0.599	0.605	0.609	0.650	0.584	0.590
	Hamming Loss	0.132	0.124	0.132	0.125	0.155	0.300	0.200	0.138	0.125
	Ranking Loss	0.159	0.140	0.151	0.143	0.178	0.405	0.304	0.145	0.125
FastText	Precision	0.882	0.905	0.901	0.908	0.884	0.752	0.894	0.896	0.903
	Avg_Precision	0.895	0.904	0.900	0.905	0.889	0.851	0.898	0.888	0.895
	Micro_Precision	0.888	0.899	0.895	0.900	0.883	0.837	0.891	0.882	0.888
	Macro_Precision	0.904	0.917	0.911	0.918	0.909	0.855	0.915	0.902	0.908
	Recall	0.880	0.891	0.889	0.893	0.853	0.762	0.869	0.885	0.888
	Micro_Recall	0.875	0.887	0.885	0.889	0.849	0.774	0.865	0.881	0.884
	Macro_Recall	0.880	0.899	0.895	0.901	0.879	0.776	0.889	0.886	0.894
	F1-score	0.887	0.898	0.895	0.899	0.870	0.797	0.883	0.886	0.891
	Micro_F1	0.881	0.893	0.890	0.894	0.865	0.796	0.878	0.881	0.886
	Macro_F1	0.853	0.875	0.872	0.878	0.843	0.723	0.859	0.861	0.870
	Acuuracy	0.775	0.802	0.803	0.807	0.735	0.561	0.768	0.784	0.800
	Subset Accuracy	0.591	0.596	0.593	0.594	0.602	0.604	0.598	0.588	0.589
	Hamming Loss	0.135	0.122	0.126	0.121	0.157	0.258	0.141	0.135	0.130
	Ranking Loss	0.168	0.137	0.138	0.132	0.183	0.378	0.161	0.145	0.135
Word2Vec	Precision	0.883	0.905	0.897	0.900	0.878	0.706	0.887	0.897	0.901
	Avg_Precision	0.895	0.903	0.904	0.908	0.895	0.761	0.898	0.889	0.900
	Micro_Precision	0.889	0.896	0.900	0.904	0.888	0.749	0.892	0.883	0.894
	Macro_Precision	0.904	0.916	0.914	0.918	0.915	0.761	0.912	0.903	0.910
	Recall	0.886	0.891	0.884	0.884	0.843	0.760	0.862	0.888	0.886
	Micro_Recall	0.881	0.886	0.880	0.881	0.839	0.790	0.858	0.884	0.881
	Macro_Recall	0.881	0.898	0.894	0.897	0.877	0.711	0.884	0.888	0.894
	F1-score	0.891	0.897	0.894	0.896	0.868	0.746	0.880	0.889	0.893
	Micro_F1	0.885	0.891	0.890	0.892	0.863	0.750	0.874	0.883	0.888
	Macro_F1	0.856	0.874	0.870	0.873	0.839	0.668	0.854	0.864	0.871
	Acuuracy	0.782	0.800	0.799	0.801	0.723	0.537	0.763	0.792	0.803
	Subset Accuracy	0.590	0.592	0.602	0.602	0.604	0.564	0.598	0.584	0.596
	Hamming Loss	0.130	0.123	0.127	0.125	0.161	0.292	0.146	0.132	0.128
	Ranking Loss	0.163	0.137	0.146	0.145	0.199	0.383	0.171	0.141	0.138

Table S15. Performance comparison of 9 adapted deep learning predictors based on 3 different embeddings over EHR-multiclass dataset

Embedding	Metrics	DL Predictor								
		Region Embedding	FastText	Text CNN	DPCNN	Text VDCNN	Attentive´ ConvNet	Text RNN	DRNN	Text RCNN
Random Embedding	Precision	0.800	0.805	0.802	0.796	0.675	0.396	0.720	0.714	0.809
	Avg_Precision	0.780	0.779	0.782	0.751	0.578	0.482	0.675	0.645	0.787
	Micro_Precision	0.506	0.475	0.530	0.435	0.129	0.232	0.249	0.206	0.510
	Macro_Precision	0.799	0.799	0.806	0.776	0.610	0.506	0.699	0.671	0.809
	Recall	0.849	0.855	0.818	0.839	0.765	0.398	0.830	0.793	0.816
	Micro_Recall	0.704	0.691	0.627	0.572	0.177	0.266	0.386	0.301	0.615
	Macro_Recall	0.785	0.788	0.789	0.772	0.627	0.416	0.697	0.679	0.794
	F1-score	0.813	0.815	0.800	0.793	0.657	0.420	0.745	0.711	0.801
	Micro_F1	0.588	0.562	0.573	0.493	0.145	0.232	0.302	0.243	0.557
	Macro_F1	0.755	0.761	0.756	0.745	0.597	0.357	0.672	0.651	0.764
	Acuuracy	0.668	0.680	0.660	0.665	0.509	0.200	0.596	0.570	0.673
	Subset Accuracy	0.154	0.149	0.154	0.137	0.087	0.140	0.144	0.118	0.160
	Hamming Loss	0.030	0.030	0.033	0.033	0.051	0.104	0.039	0.044	0.033
	Ranking Loss	0.208	0.207	0.202	0.229	0.392	0.524	0.305	0.332	0.198
FastText	Precision	0.798	0.808	0.796	0.773	0.652	0.298	0.708	0.701	0.804
	Avg_Precision	0.782	0.779	0.775	0.749	0.573	0.400	0.662	0.649	0.781
	Micro_Precision	0.517	0.485	0.489	0.432	0.143	0.192	0.231	0.199	0.482
	Macro_Precision	0.802	0.799	0.798	0.770	0.601	0.406	0.682	0.670	0.803
	Recall	0.834	0.861	0.823	0.806	0.804	0.330	0.837	0.857	0.810
	Micro_Recall	0.682	0.688	0.664	0.535	0.195	0.185	0.360	0.334	0.556
	Macro_Recall	0.784	0.790	0.783	0.758	0.613	0.324	0.682	0.674	0.789
	F1-score	0.807	0.818	0.799	0.776	0.668	0.346	0.738	0.738	0.795
	Micro_F1	0.588	0.568	0.561	0.476	0.162	0.183	0.280	0.248	0.515
	Macro_F1	0.754	0.763	0.751	0.729	0.586	0.269	0.657	0.651	0.759
	Acuuracy	0.663	0.682	0.660	0.645	0.506	0.125	0.584	0.583	0.670
	Subset Accuracy	0.158	0.152	0.160	0.154	0.098	0.179	0.137	0.130	0.157
	Hamming Loss	0.032	0.029	0.033	0.037	0.048	0.141	0.040	0.039	0.034
	Ranking Loss	0.206	0.206	0.209	0.237	0.401	0.620	0.321	0.333	0.203
Word2Vec	Precision	0.803	0.806	0.791	0.779	0.649	0.266	0.718	0.708	0.809
	Avg_Precision	0.782	0.779	0.772	0.759	0.563	0.247	0.671	0.660	0.786
	Micro_Precision	0.502	0.483	0.483	0.459	0.123	0.120	0.234	0.225	0.488
	Macro_Precision	0.801	0.799	0.793	0.778	0.594	0.253	0.693	0.680	0.806
	Recall	0.855	0.854	0.825	0.820	0.793	0.550	0.820	0.842	0.815
	Micro_Recall	0.717	0.681	0.652	0.606	0.195	0.267	0.360	0.368	0.614
	Macro_Recall	0.788	0.789	0.778	0.764	0.607	0.251	0.694	0.682	0.794
	F1-score	0.817	0.814	0.797	0.788	0.656	0.325	0.738	0.740	0.800
	Micro_F1	0.590	0.564	0.553	0.522	0.146	0.160	0.283	0.279	0.543
	Macro_F1	0.761	0.761	0.748	0.735	0.579	0.233	0.669	0.658	0.765
	Acuuracy	0.678	0.678	0.660	0.647	0.497	0.180	0.598	0.587	0.681
	Subset Accuracy	0.155	0.152	0.160	0.157	0.093	0.069	0.136	0.139	0.155
	Hamming Loss	0.030	0.030	0.033	0.034	0.050	0.084	0.040	0.039	0.033
	Ranking Loss	0.206	0.207	0.214	0.228	0.408	0.750	0.310	0.323	0.200

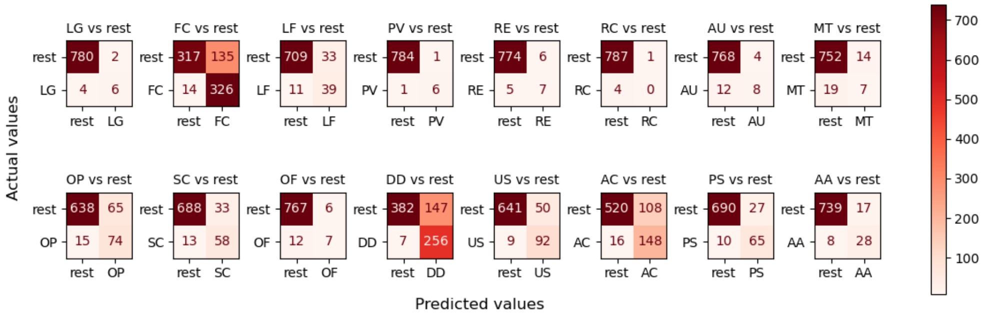

(a) OkapiBM25-LP-LR

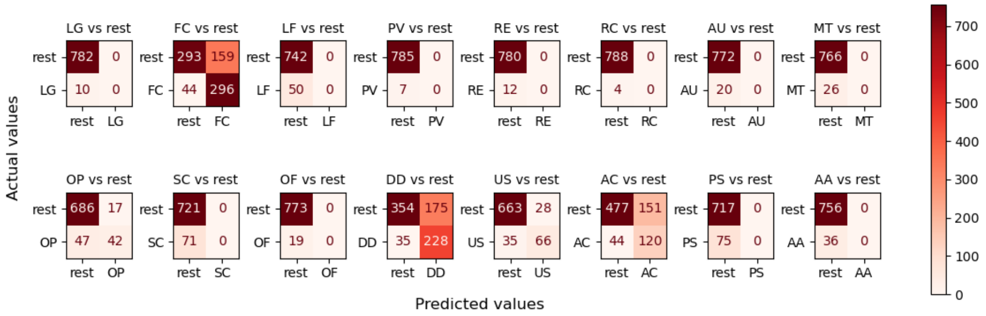

(b) OkapiBM25-MLKNN

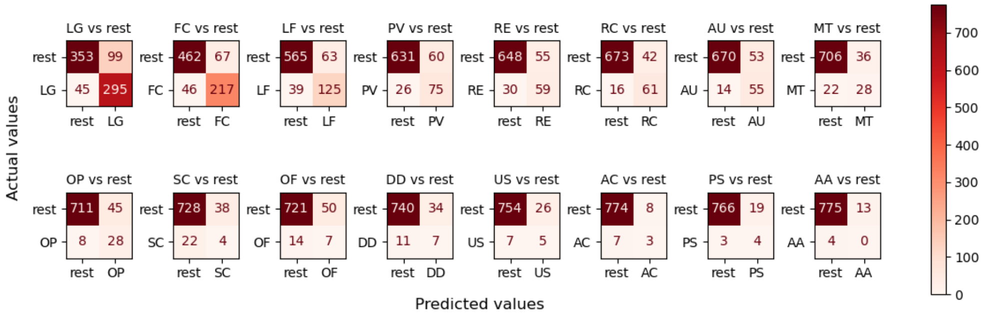

(c) FastText-TextRCNN

Figure S1. Class-wise performance analysis of MLR and baseline predictors in terms of number of correct and wrong predictions over promise dataset

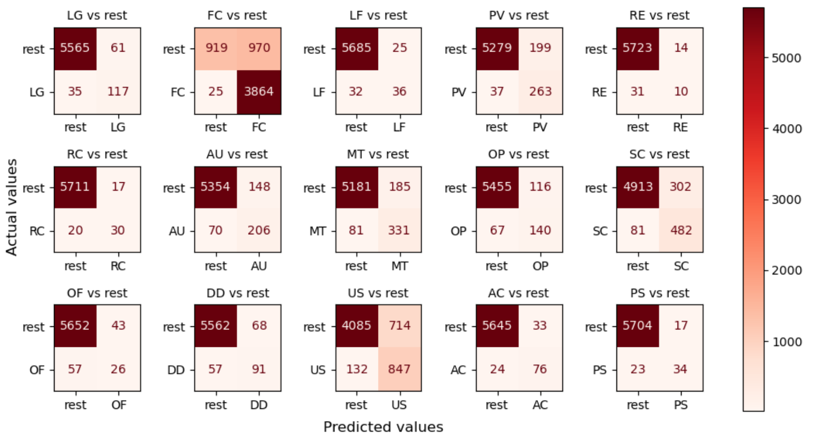

(a) OkapiBM25-LP-LR

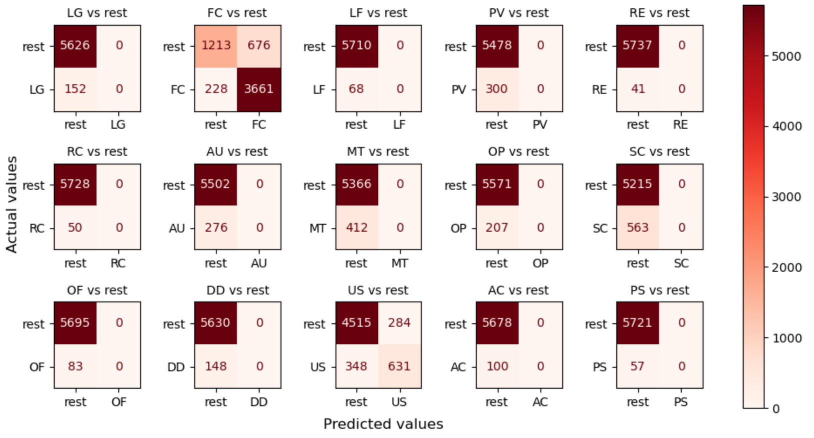

(b) OkapiBM25-MLKNN

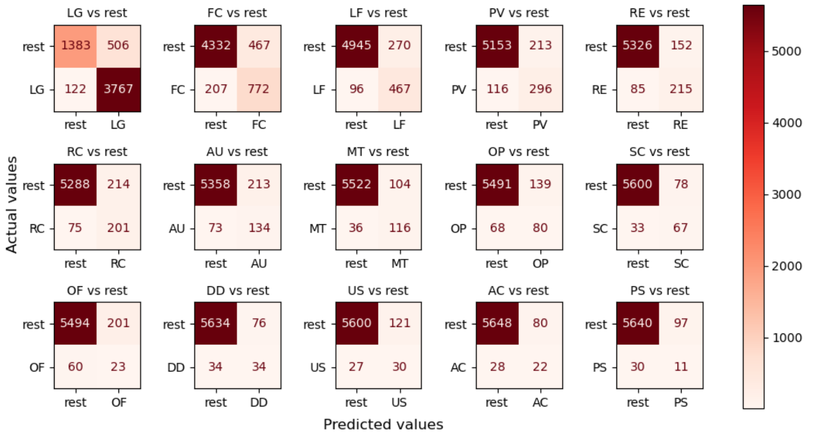

(c) FastText-TextRCNN

Figure S2. Class-wise performance analysis of MLR and baseline predictors in terms of number of correct and wrong predictions over EHR-Multiclass dataset

REFERENCES

[1]Y. Kim, Convolutional neural networks for sentence classification, in: Proceedings of the 2014 Conference on Empirical Methods in Natural Language Processing (EMNLP), Association for Computational Linguistics, Doha, Qatar, 2014, pp. 1746–1751. doi:10.3115/v1/D14-1181. URL https://aclanthology.org/D14-1181

[2]A. Haghighat, A. Sharma, A computer vision-based deep learning model to detect wrong-way driving using pan–tilt–zoom traffic cameras, Computer-Aided Civil and Infrastructure Engineering 38 (1) (2023) 119–132.

[3]I. N. Khasanah, Sentiment classification using fasttext embedding and deep learning model, Procedia Computer Science 189 (2021) 343–350.

[4]P. Baldi, P. J. Sadowski, Understanding dropout, Advances in neural information processing systems 26 (2013).

[5]S. Liu, Z. Liu, Multi-channel cnn-based object detection for enhanced situation awareness, arXiv preprint arXiv:1712.00075 (2017).

[6]R. Johnson, T. Zhang, Deep pyramid convolutional neural networks for text categorization, in: Proceedings of the 55th Annual Meeting of the Association for Computational Linguistics (Volume 1: Long Papers), 2017, pp. 562–570.

[7]S. Yu, D. Liu, Y. Zhang, S. Zhao, W. Wang, Dptcn: A novel deep cnn model for short text classification, Journal of Intelligent & Fuzzy Systems 41 (6) (2021) 7093–7100.

[8]A. Conneau, H. Schwenk, L. Barrault, Y. Lecun, Very deep convolutional networks for text classification, arXiv preprint arXiv:1606.01781 (2016).

[9]K. He, X. Zhang, S. Ren, J. Sun, Deep residual learning for image recognition, in: Proceedings of the IEEE conference on computer vision and pattern recognition, 2016, pp. 770–778.

[10]K. Simonyan, A. Zisserman, Very deep convolutional networks for large-scale image recognition, arXiv preprint arXiv:1409.1556 (2014).

[11]W. Yin, H. Schütze, Attentive convolution: Equipping cnns with rnn-style attention mechanisms, Transactions of the Association for Computational Linguistics 6 (2018) 687–702.

[12]L. Liu, F. Mu, P. Li, X. Mu, J. Tang, X. Ai, R. Fu, L. Wang, X. Zhou, Neuralclassifier: an open-source neural hierarchical multi-label text classification toolkit, in: Proceedings of the 57th Annual Meeting of the Association for Computational Linguistics: System Demonstrations, 2019, pp. 87–92.

[13]W. Yin, D. Roth, Term definitions help hypernymy detection, arXiv preprint arXiv:1806.04532 (2018).

[14]S. Narayan, The generalized sigmoid activation function: Competitive supervised learning, Information sciences 99 (1-2) (1997) 69–82.

[15]B. Karlik, A. V. Olgac, Performance analysis of various activation functions in generalized mlp architectures of neural networks, International Journal of Artificial Intelligence and Expert Systems 1 (4) (2011) 111–122.

[16]P. Liu, X. Qiu, X. Huang, Recurrent neural network for text classification with multi-task learning, arXiv preprint arXiv:1605.05101 (2016).

[17]B. Wang, Disconnected recurrent neural networks for text categorization, in: Proceedings of the 56th Annual Meeting of the Association for Computational Linguistics (Volume 1: Long Papers), 2018, pp. 2311–2320.

[18]S. Lai, L. Xu, K. Liu, J. Zhao, Recurrent convolutional neural networks for text classification, in: Proceedings of the AAAI conference on artificial intelligence, Vol. 29, 2015.

[19]C. Qiao, B. Huang, G. Niu, D. Li, D. Dong, W. He, D. Yu, H. Wu, A new method of region embedding for text classification., in: ICLR (Poster), 2018.

[20]A. Joulin, E. Grave, P. Bojanowski, T. Mikolov, Bag of tricks for efficient text classification, arXiv preprint arXiv:1607.01759 (2016).
